# Influenza A viruses use multivalent sialic acid clusters for cell binding and receptor activation

**DOI:** 10.1371/journal.ppat.1008656

**Published:** 2020-07-08

**Authors:** Christian Sieben, Erdinc Sezgin, Christian Eggeling, Suliana Manley

**Affiliations:** 1 Laboratory of Experimental Biophysics, Institute of Physics, École Polytechnique Fédérale de Lausanne (EPFL), Route Cantonale, Lausanne, Switzerland; 2 MRC Human Immunology Unit, University of Oxford, MRC Weatherall Institute of Molecular Medicine, Headley Way, Oxford, United Kingdom; University of Wisconsin-Madison, UNITED STATES

## Abstract

Influenza A virus (IAV) binds its host cell using the major viral surface protein hemagglutinin (HA). HA recognizes sialic acid, a plasma membrane glycan that functions as the specific primary attachment factor (AF). Since sialic acid alone cannot fulfill a signaling function, the virus needs to activate downstream factors to trigger endocytic uptake. Recently, the epidermal growth factor receptor (EGFR), a member of the receptor-tyrosine kinase family, was shown to be activated by IAV and transmit cell entry signals. However, how IAV’s binding to sialic acid leads to engagement and activation of EGFR remains largely unclear. We used multicolor super-resolution microscopy to study the lateral organization of both IAV’s AFs and its functional receptor EGFR at the scale of the IAV particle. Intriguingly, quantitative cluster analysis revealed that AFs and EGFR are organized in partially overlapping submicrometer clusters in the plasma membrane of A549 cells. Within AF domains, the local AF concentration reaches on average 10-fold the background concentration and tends to increase towards the cluster center, thereby representing a multivalent virus-binding platform. Using our experimentally measured cluster characteristics, we simulated virus diffusion on a flat membrane. The results predict that the local AF concentration strongly influences the distinct mobility pattern of IAVs, in a manner consistent with live-cell single-virus tracking data. In contrast to AFs, EGFR resides in smaller clusters. Virus binding activates EGFR, but interestingly, this process occurs without a major lateral EGFR redistribution, indicating the activation of pre-formed clusters, which we show are long-lived. Taken together, our results provide a quantitative understanding of the initial steps of influenza virus infection. Co-clustering of AF and EGFR permit a cooperative effect of binding and signaling at specific platforms, thus linking their spatial organization to their functional role during virus-cell binding and receptor activation.

## Author summary

The plasma membrane is the major interface between a cell and its environment. This complex and dynamic organelle needs to protect, as a barrier, but also transmit subtle signals into and out of the cell. For the enveloped virus IAV, the plasma membrane represents both a major obstacle to overcome during infection, and the site for the assembly of progeny virus particles. However, the organisation of the plasma membrane–a key to understanding how viral entry works—at the scale of an infecting particle (length scales < 100 nm) remains largely unknown. Sialylated glycans serve as IAV attachment factors but are not able to transmit signals across the plasma membrane. Receptor tyrosine kinases were identified to be activated upon virus binding and serve as functional receptors. How IAV engages and activates its functional receptors while initially binding glycans still remains speculative. Here, we use super resolution microscopy to study the lateral organization of plasma membrane-bound molecules involved in IAV infection, as well as their functional relationship. We find that molecules are organized in submicrometer nanodomains and, in combination with virus diffusion simulations, present a mechanistic model for how IAV first engages with AFs in the plasma membrane to subsequently engage and trigger entry-associated membrane receptors.

## Introduction

Influenza A viruses (IAV) cause severe respiratory tract infections in humans, often leading to seasonal local epidemics as well as periodic global pandemics [1]. During cell binding, IAV engages with low affinity attachment factors (AFs) as well as functional receptors to trigger cell entry by endocytosis. However, little is known about the lateral co-organization of both at the scale of an infecting virus, and how their organization relates to their functional role during virus infection.

**Fig 1:**
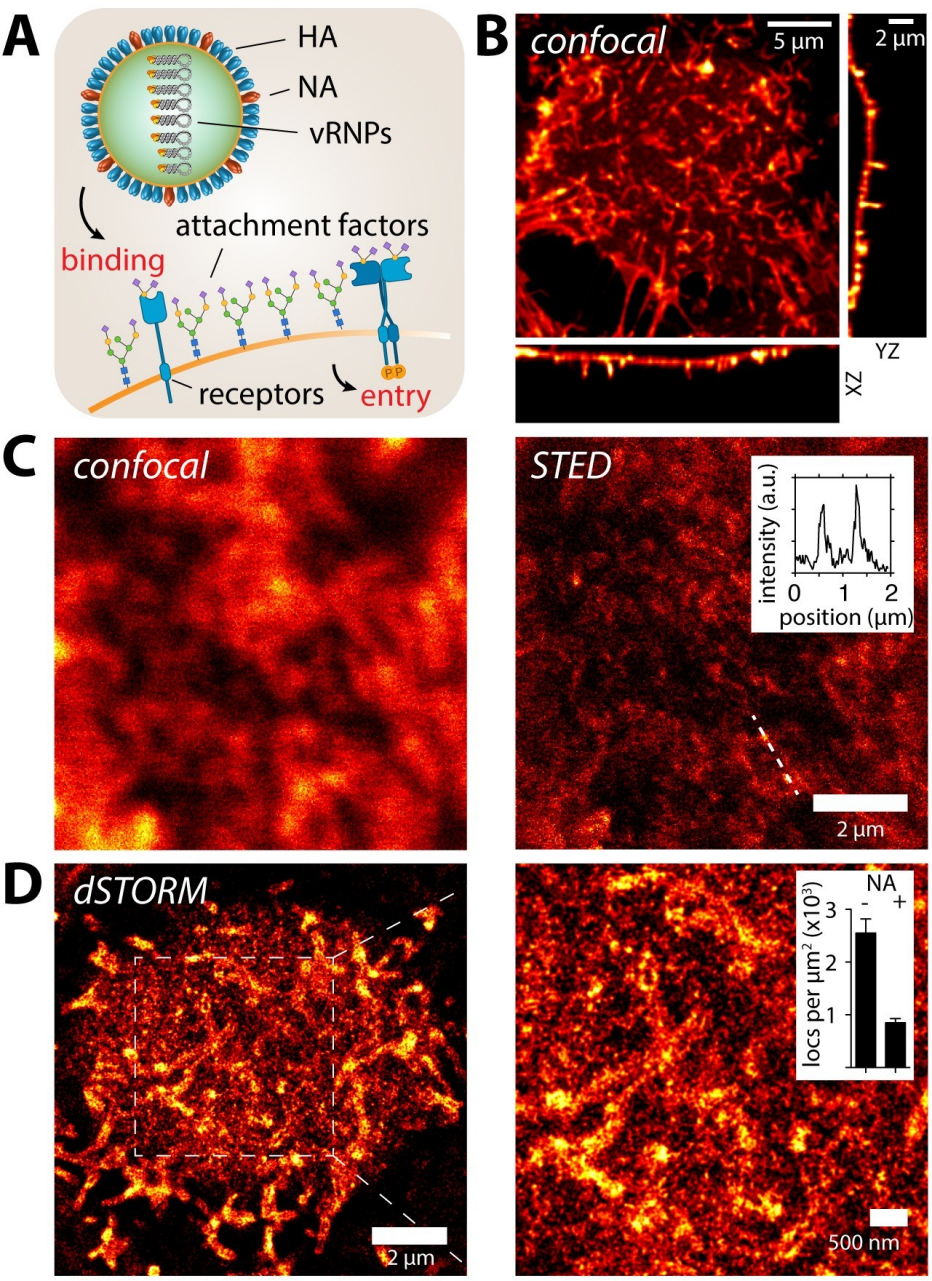
Sialylated IAV attachment factors are organized in nanodomains on A549 cells. (**A**) Influenza virus is an enveloped particle that encapsulates its segmented (-)vRNA genome built of 8 viral ribonucleoprotein complexes (vRNPs). The viral membrane harbors the two glycoproteins hemagglutinin (HA) and neuraminidase (NA). HA is responsible for binding sialic acid (SA) containing attachment factors on the host cell plasma membrane. Upon cell-binding, the virus needs to activate functional receptors to trigger endocytosis. (**B**) Confocal imaging of live A549 cells labelled with SNA (conjugated to JF549). The cells feature a non-uniform SNA distribution across the plasma membrane. Large finger-like protrusions can be observed on the dorsal side of the cell. (**C**) Confocal and STED live-cell imaging of A549 cells labelled with SNA (conjugated to StarRed) confirms the existence of finger-like protrusions as well as a population of smaller nanodomains with diameter of ~100 nm (**C**, right, inset). (**D**) To increase the resolution and focus on the small nanodomain we performed STORM imaging of A549 cells labelled with SNA (conjugated to Alexa647). Reconstructed STORM images confirm two major structural features (1) finger-like protrusions as well as (2) small nanodomains. Cell treatment with neuraminidase (NA, 0.01 U/ml for 2h) led to a strong reduction of the localization density due to the cleavage and hence decrease local concentration of SA (**D**, right, inset).

The viral hemagglutinin (HA), a trimeric glycoprotein that covers ~ 90% of the viral surface, mediates the initial IAV-cell contact (Fig 1A) [2]. The most common AF for IAV is N-acetylneuraminic, or sialic, acid (SA), a highly abundant cell-surface glycan that within its glyosidic linkage encodes IAV species specificity. Human-pathogenic IAV strains preferentially bind α-2,6-linked SA, while avian-pathogenic viruses prefer to bind α-2,3-linked SA. This specificity can be attributed to the topology of the glycans, which more readily form contacts with receptor-binding domains of complementary HAs [3]. A common feature of glycan-protein interactions is their low affinity, which for HA-sialic acid lies in the millimolar range [4] and should make it challenging for the virus to form stable interactions with cells. Although AFs are highly abundant, which could lead to stable adhesion, single-virus tracking showed that IAVs have some freedom to explore the cell surface [5–7]. Indeed, it remains largely unclear how an initial low-affinity interaction leads to a stable and specific but also dynamic virus-cell contact enabling a successful infection.

After binding, IAVs enter cells by receptor-mediated endocytosis, where clathrin-mediated endocytosis was shown to constitute the major [7], albeit not the only route [8]. Since AFs cannot trigger endocytosis, an active signal-processing receptor must be engaged to allow viral entry. Receptor-tyrosine kinases were shown to be able to fulfill this function [9]. Specifically, it was shown that, among other receptor-tyrosine kinases, epidermal growth factor receptor (EGFR) was activated during and required for efficient IAV cell entry. However, how IAV finds and activates EGFR has remained speculative. While molecular and structural information is available for both HA-SA [3] and EGFR-EGF interactions [10], much less is known about their spatial co-organization within the plasma membrane, and how it could enable EGFR activation during IAV cell infection.

Over the last decades, electron microscopy has provided a detailed structural picture of isolated influenza virus particles [2] as well as their individual proteins [11]. However, imaging and quantitative analysis of heterogeneous cellular structures and their dynamics at the nanoscale remains challenging. Super-resolution microscopy represents an excellent tool to study cellular organization at the scale of an infecting viral particle (<100 nm) [12]. Here, we combined two complementary approaches, which together provide a versatile toolbox to meet this challenge [12,13]. We used single molecule localization microscopy (SMLM) techniques known as stochastic optical reconstruction microscopy (STORM) and (fluorescence) photoactivated localization microscopy ((f)PALM) together with live-cell stimulated emission depletion (STED) [14] microscopy to image molecular organization and track single molecules within the plasma membrane of IAV host cells [15–17].

We quantitatively analyzed the spatial organization of IAV AFs as well as EGFR on the surface of human alveolar epithelial cells. We found that AFs are organized in virus-sized clusters featuring a density gradient that decreases from a dense central core to the periphery. Using their experimentally-determined characteristics, we used simulations to investigate their role in virus-cell interactions. These simulations are in good agreement with live-cell virus tracking experiments, and together, they suggest that the spatial organization of AFs largely influences virus-cell interactions during the early phase of virus infection. We further show that AF nanodomains overlap nonrandomly with EGFR clusters thereby promoting AF-mediated EGFR activation. Interestingly, our results further suggest that pre-existing EGFR clusters are responsible for IAV-mediated receptor activation. We provide a novel view on the initial events of influenza virus infection and offer new insights into the functional role of the spatial organization of cell surface AFs and receptors.

## Results

### Sialic acid-containing IAV attachment factors are organized in nanodomains on the plasma membrane of A549 cells

To examine the spatial organization of IAV AFs within the plasma membrane of permissive epithelial cells, we sought to selectively label sialic acid moieties which are preferably recognized by human-pathogenic IAVs, such as H3N2/X31 [4] used here. For this purpose, we used the plant lectin *Sambuccus nigra* agglutinin (SNA), which labels α-2,6-linked SA, to serve as a primary IAV AF label (S1 Text, S1 Fig). Using confocal microscopy, we found that SNA strongly labelled the plasma membrane of live A549 cells (Fig 1B), showing inhomogeneous staining and enrichment in finger-like protrusions that morphologically appeared to be microvilli. We then used STED microscopy to more carefully study the smoother regions of the plasma membrane between the microvilli. On live A549 cells, we detected a strong heterogeneity of SNA cell surface labelling including small clusters at the scale of 100–200 nm (Fig 1C, right panel, inset).

Our next goal was to investigate the lateral organization of IAV AFs at the scale of the virus-cell interface, in fixed cells to avoid motion blurring. We initially confirmed that our cell fixation protocol successfully immobilized plasma membrane molecules (S2 Fig), then performed STORM and STED in parallel to image the plasma membrane at the scale of small spherical H3N2/X31 virions (average diameter of 120 nm [2]). STORM (Fig 1D) and STED (S3A Fig) imaging of fixed A549 cells supported our observations made on live cells that SNA stains a variety of small nanostructures on the flat parts of the plasma membrane. We also found a similar distribution on MDCK cells, which are highly susceptible for IAV (S3B Fig). As an SNA-independent control, we used wheat germ agglutinin (WGA), a general SA-binding lectin. WGA labelled A549 cells at higher density, while again highlighting microvilli and nanoscale clusters (S3C Fig). Finally, we labeled cells using antibodies against ezrin, an actin-binding protein. Ezrin is highly enriched in microvilli [18], and confirmed that the large finger-like structures are indeed microvilli (S4A Fig). Since microvilli are not actively involved in endocytosis [19,20], we focused on the population of smaller AF clusters.

### Quantitative analysis of SNA nanodomains

**Fig 2:**
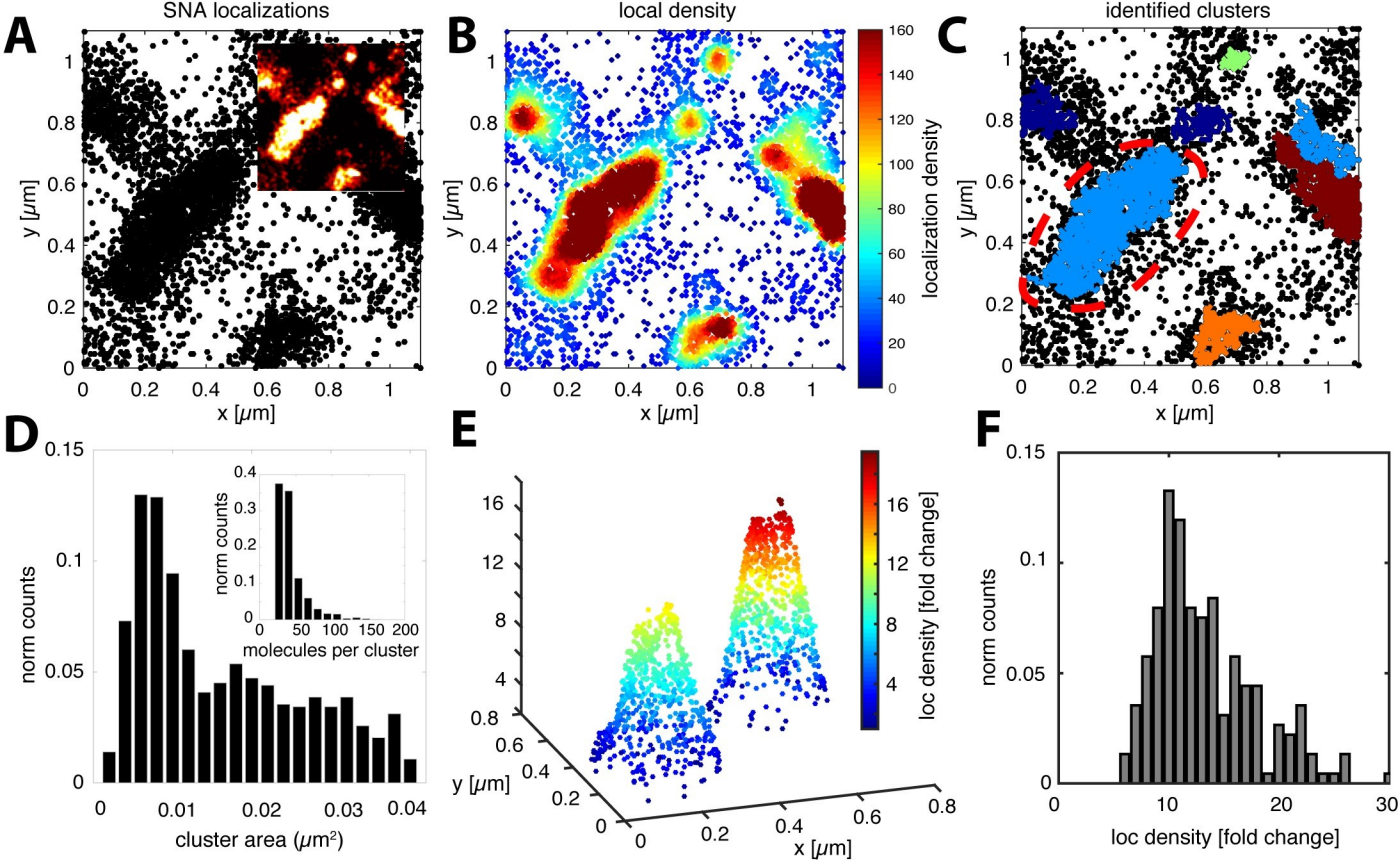
Density-based localization analysis reveals small SA clusters between microvilli. (**A**) Spatial distribution of STORM localizations from SNA-A647 on A549 cells showing the coexistence of two structural features, (1) large microvilli as well as (2) small nanodomains. The inset in **A** shows a pixel image reconstruction (10 nm/pxl) of the localization map in **A**. (**B)** Density distribution of localizations shown in **A** within a search radius of 50 nm. Color scale according to number of neighbor localizations. (**C**) Final clustering result with identified clusters allows quantification of the cluster area. After all identified clusters were filtered according to their size to selectively analyze non-microvilli structures, we found clusters with an area between 0.005–0.04 μm^2^ (**D**). The large cluster labelled by the red dashed line was filtered out (see also S4 Fig). Distribution of the number of molecules per cluster as estimated according to the number of localizations (**D**, inset). The inner structure of non-microvilli clusters was analyzed according to their local localization density (**B**). (**E**) Representative example of two identified clusters showing their inner density gradient. The color code represents the number of nearest neighbor localizations within a radius of 30 nm (i.e. the local localization density). The localization density is plotted on the vertical axis. Distribution of the density difference between background and the cluster center over all identified cluster (**F**).

To quantify the lateral organization of AFs from our STORM localization data, we used an algorithm based on the detection of local density differences. To facilitate the analysis, we developed a workflow (see Methods) that allowed us to extract geometrical properties of the clusters as well as an estimate of the number of AF molecules (Fig 2A–2C). To identify and exclude the large microvilli cluster population, we first performed cluster identification on the ezrin localization maps (S4B Fig). We found that the large ezrin clusters had dimensions of 10–50 nm across the short and 0.5–2 μm along the long axis (S4B Fig). These parameters were then used to filter out the large cluster population corresponding to microvilli identified in SNA localization maps (Fig 2C). After filtering, we identified a heterogeneous population of small clusters with a mean area of 0.016 μm^2^ (Fig 2D). Since the cluster area was at the same scale as the projected two-dimensional area of a spherical IAV (0.0079 μm^2^ for r = 50 nm), we took a closer look at the density of molecules within each cluster. To define a local density, we counted the number of nearest neighbor localizations within a distance of three times the localization precision (3σ ~ 30 nm) (S5 Fig). Interestingly, we found that AF clusters have an average 10-fold enrichment compared to the local background while some reach an even 20-fold increase (Fig 2E and 2F). As a control, we used simulations to test the effect of localization precision on local concentration; however, this accounts at most for an enrichment of < 8-fold (S6D Fig).

Following the identification of AF domains, we wanted to understand whether their observed organization would have an impact on virus binding compared to a homogeneous distribution. We constructed a virus binding simulation in which we gradually redistribute AF molecules from a random into a clustered organization, keeping the total number of molecules constant. In parallel, we simulated a stable population of clusters within a background of free individual molecules and vary the number of molecules per cluster. In both cases, we simulate a spherical virus particle and project its size as a landing spot on the simulated molecule surface. Binding is counted as successful if the virus contacts at least 10 AF molecules. As shown in S7 Fig, we find a positive correlation between AF clustering and virus binding indicating that nanoclustering enhances the probability of efficient virus adhesion.

### Single-IAV tracking indicates exploratory motion affected by the local AF concentration

**Fig 3:**
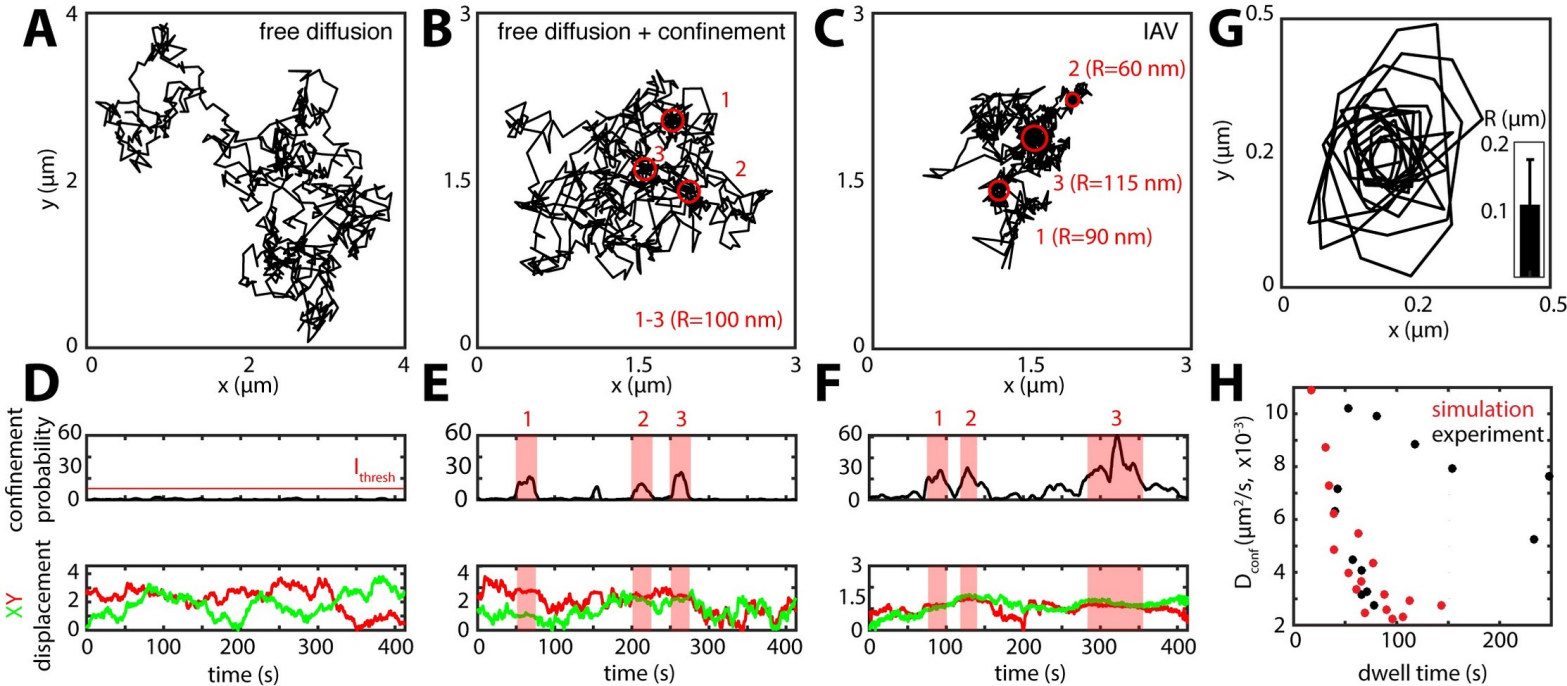
IAV performs a receptor concentration-driven random walk on the plasma membrane. Based on our quantitative analysis of the AF distribution on A549 cells, we hypothesize a motion behavior that is driven by the local AF concentration. We simulate this behavior initially as a 2D random walk with free diffusion coefficient D_free_ (**A**). (**B**) Next, we simulate AF clusters (red circles, r = 100 nm), which would, due to the increased SA concentration, lead to a temporal confinement (D_conf_ < D_free_). To identify confined regions within the simulated virus trajectories, we establish a confinement probability I_conf_. Accordingly, a free diffusing particle shows only fluctuation in of I_conf_ (**D**), while the addition of temporal confinement leads to a clear increase that overlaps with stationary phases of the particle as visible in the XY displacement plot (**E**). We used the confinement probability to analyze experimental virus trajectories in particular the mixed type of trajectories (**C**) (see also S8 Fig) (**C**). I_conf_ shows a clear signature of temporal confinement (**F**) similar to the model prediction (**E**). As a further challenge for our model, we performed a subtrajectory analysis, thereby extracting the dwell time, D_conf_ as well as the area of the respective temporal confinement in our virus trajectories. (**G**) shows an overlay of the perimeters of the extracted confined regions as well as the average radius (R). From our simulated data, correlation of D_conf_ with the dwell time shows that a local increase in AF concentration (i.e. decreased diffusion) due to the encounter of an SA nanodomain leads to an increased local dwell time (**H**, red markers). We observed a similar behavior, when we tested the confinement dwell time in experimental virus trajectories (**H**, black markers).

We wondered how IAV motion on the cell membrane is affected by AF clustering. To develop a hypothesis and custom analysis, we first established a simple diffusion model to simulate the behavior of individual viruses on a flat part of the cell surface. The model assumes a two-dimensional random walk [21] where the virus undergoes periods of free diffusion (with diffusion coefficient *D*_*free*_) until it encounters a region of high AF concentration and becomes transiently confined (with diffusion coefficient *D*_*conf*_). The time a simulated particle stays confined will depend on *D*_*conf*_ and the size of the confined region (i.e. the AF cluster size as measured using STORM). To identify and quantify confined regions, we used a previously published confinement probability *I*_*conf*_ denoting the probability for the particle to be confined at time *t* [22]. Although even simulations of free diffusion displayed periods of apparent confinement, due to the stochastic nature of thermal motion (S8A Fig), the simulations revealed different characteristic *I*_*conf*_ signatures for free (Fig 3A and 3D) versus confined diffusion (Fig 3B and 3E, S8 Fig and S1 Movie). Finally, we defined a threshold level of confinement, *I*_*thresh*_ *= 5*, to exclude these random fluctuations and identify confined areas and confinement dwell times (Fig 3A and 3D; S8 Fig).

To test whether our model is consistent with the motion of IAV, we performed single-virus tracking on live A549 cells. We considered physiological conditions (37°C) as well as conditions that suppress virus endocytosis (4°C, dynasore treatment) to prolong the particles’ residence time on the cell surface. The trajectory analysis revealed different modes of movement, which we classified into four types: (1) confined, (2) ballistic, (3) drifting and (4) mixed (S8 Fig). The ballistic movement is directional, at speeds up to 1–2 μm/sec; thus, we assigned it to microtubule-associated transport as previously described [6,7]. Since this type of movement follows a successful virus internalization, we expected to see it decrease in frequency after blocking endocytosis. Indeed, the fraction of ballistic trajectories dropped from 30% to below 5% when we imaged at low temperature or in the presence of 40 μM dynasore [23]. Interestingly, we observed a marked increase of the other three motion classes, suggesting that they take place at the plasma membrane (S9 Fig). When we then took a closer look at the mixed class of trajectories, we found regions of extended IAV residence time indicating spatial confinement alternating with free diffusion (Fig 3C). Consequently, we applied our confinement analysis to the mixed IAV trajectory class, which revealed areas of pronounced confinement that did indeed alternate with regions of free diffusion (Fig 3F).

If AF domains of different lateral concentrations coexist in the plasma membrane, as observed using STORM, these regions could serve as binding platforms for diffusing viruses. According to our simple diffusion model, the local AF concentration dominates *D*_*conf*_ (see Methods), which in turn determines the particles dwell time inside the confined regions. To test in experiments whether *D*_*conf*_ does negatively correlate with the dwell time, we performed a subtrajectory analysis where each trajectory was screened for confinement according to *I*_*conf*_. For each confined region, we then extracted the dwell time as well as *D*_*conf*_ (see Methods), finding that they are indeed inversely related as predicted by our diffusion model (Fig 3H). Subtrajectory analysis further allowed us to estimate the spatial dimensions of the confined regions (Fig 3G). We found an average radius of 104 nm corresponding to a median area of 0.057 μm^2^, which is only slightly larger than the SNA cluster size found using STORM (Fig 2D). In summary, the lateral organization of AFs, characterized using STORM, has a predicted effect on virus mobility, consistent with live-cell IAV tracking data.

### EGFR is organized in nanoclusters that non-randomly overlap with SNA domains

How does IAV-glycan binding translate into cell entry? To study the nanoscale spatial relationship between AFs and functional receptors, we focused on the previously identified IAV receptor EGFR [9,24]. Initially, we verified that our IAV strain (H3N2/X31) binds to and depends on EGFR for cell infection in our experimental system. We found that about 20% of IAVs colocalized with EGFR (S10 Fig), and that efficient infection depended on available plasma membrane EGFR and could be inhibited by EGFR-specific kinase inhibitors (S1 Fig, S11 Fig). We then investigated the lateral organization of EGFR in A549 cells using fluorescently labelled anti-EGFR antibodies. In our infection experiments, the cells were pre-incubated with serum-free infection medium (30 min) before viruses were added, a common procedure for influenza virus infection. To reproduce these conditions, we also performed a serum-free pre-incubation before the cells were fixed and immunolabelled for EGFR.

**Fig 4:**
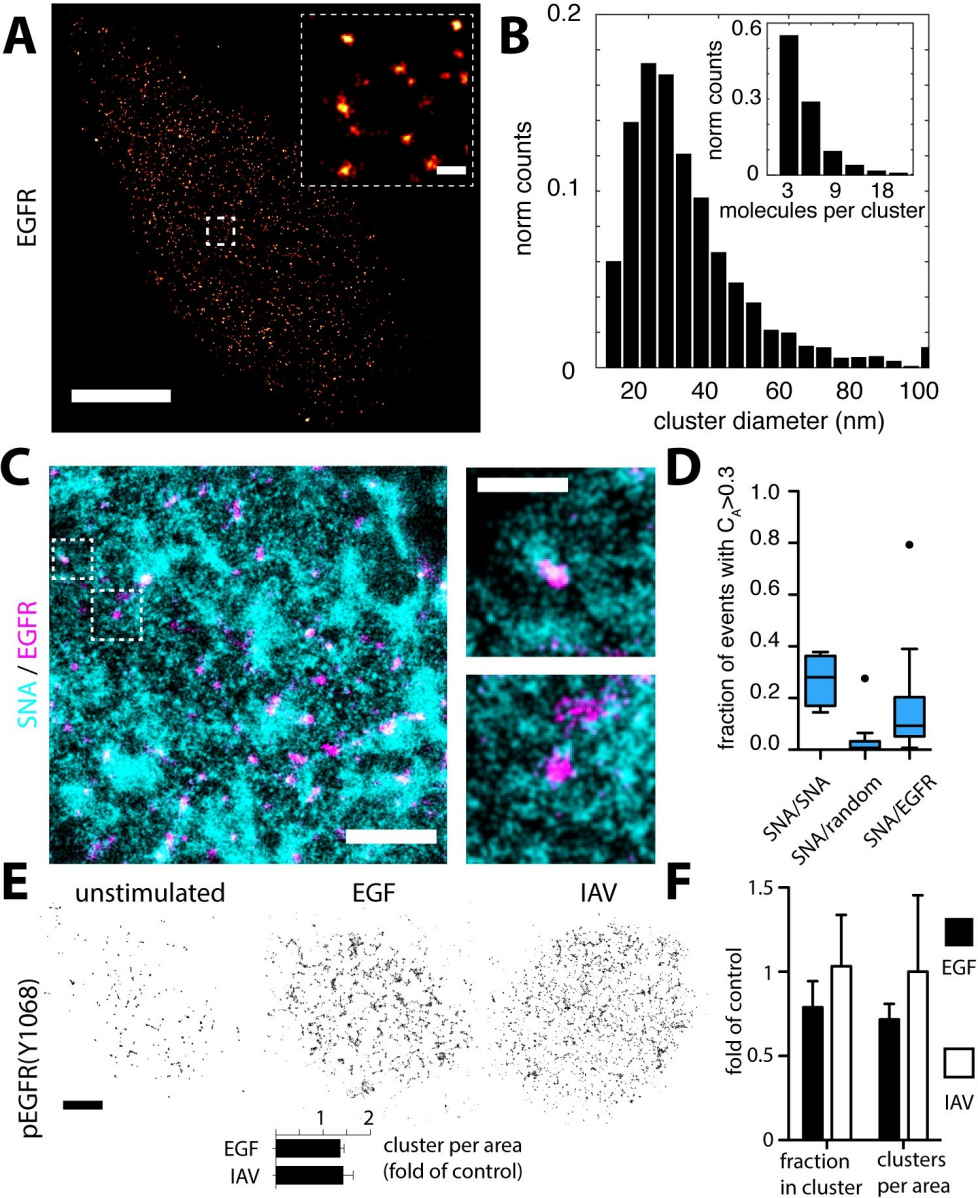
EGFR is organized in nanodomains that overlap with SNA domains. (**A**) A549 cells were labelled with antibodies against EGFR. STORM imaging revealed a clustered organization of EGFR. The clusters have an average diameter of 60 nm and contain about 5–10 molecules (**B**). Scale bar 1 μm. Inset: 100 nm. (**C**) Two-color STORM imaging of A549 cells labelled with SNA and anti-EGFR antibodies. The two panels on the right show larger magnification of the boxed areas in the left panel. Scale bars: 500 nm (left panel), 200 nm (right panel). The degree of colocalization was quantified using coordinate-based colocalization, where each localization is associated with a colocalization value C_A_. (**D**) Box plots of C_A_ distribution of SNA localizations when colocalized with (1) SNA, (2) a random distribution of localizations at equal density as EGFR and (3) EGFR. After IAV-stimulation (MOI~100), we found that phosphorylated EGFR (Y1068) is also localized in nanodomains. Although a small population of clusters seems to be phosphorylated without stimulus, we observed an increase in the activated cluster population after stimulation with IAV (MOI~100) or EGF (100 ng/ml) (**E**, lower panel). To test for a potential redistribution of EGFR, we looked at the entire population after stimulation. While after EGF stimulation, we could observe a reduction of the clustered protein fraction as well as the cluster density per area, we could not detect such a protein redistribution after IAV stimulation (**F**).

Using STORM imaging, we found that EGFR is present at much lower concentrations on the cell surface compared to SA-conjugated AFs, but interestingly, also localized in nanodomains (Fig 4A). Because of the sparsity of EGFR labeling, which might lead to false appearance of protein clusters due to fluorophore blinking [25], we established an adapted analysis scheme based on the photophysical characterization of the fluorescent probe used in our experiments (S12 Fig).

After blink correction and cluster identification, we found that in the absence of EGF stimulation 30–60% of EGFR molecules reside in clusters, which were sensitive to actin- and lipid raft destabilizing drugs (S11E Fig). The EGFR clusters had an average diameter of 29 nm and were composed of 6 molecules (Fig 4B), consistent with previous estimates using electron microcopy [26] and FRET [27]. Since IAVs bind to AFs on the cell surface, we hypothesized that SNA and EGFR should occupy the same regions on the plasma membrane to enable IAV-EGFR interactions. To test this hypothesis, we performed two-color STORM imaging (Fig 4C) using A549 cells co-labeled with SNA and anti-EGFR antibodies. Indeed, we found that EGFR clusters overlap with SNA-labeled membrane domains. However, since sialylated AFs are much more abundant than EGFRs, their colocalization could occur simply by chance. To examine this possibility, we analyzed our data using coordinate-based colocalization (CBC) [28], which estimates the spatial correlation of two localization datasets (*i*.*e*. co-clustering), reflected in the colocalization parameter *C*_*A*_. To contextualize the extent of colocalization in our SNA/EGFR dataset, we performed a positive control experiment using SNA conjugated with two different fluorophores (denoted SNA/SNA). As a negative control and to take differences in molecule density into account, for each two-color field of view in SNA/EGFR, we simulated a random dataset at the same density (denoted SNA/random). Finally, we define localizations with C_A_>0.3 as colocalized (annotated C_A>0.3_ below) (S13 Fig). As shown in Fig 4D, the negative control SNA/random reaches C_A>0.3_ = 0.035, a baseline level only accounting for random colocalization, while the experimental positive control SNA/SNA reaches a much higher value, C_A>0.3_ = 0.27. CBC analysis of SNA/EGFR colocalization reached an intermediate value, C_A>0.3_ = 0.17. These analyses reveal that the spatial overlap between EGFR and SNA is not complete and yet also not random, indicating that both molecules share a common population of membrane compartments.

**Fig 5:**
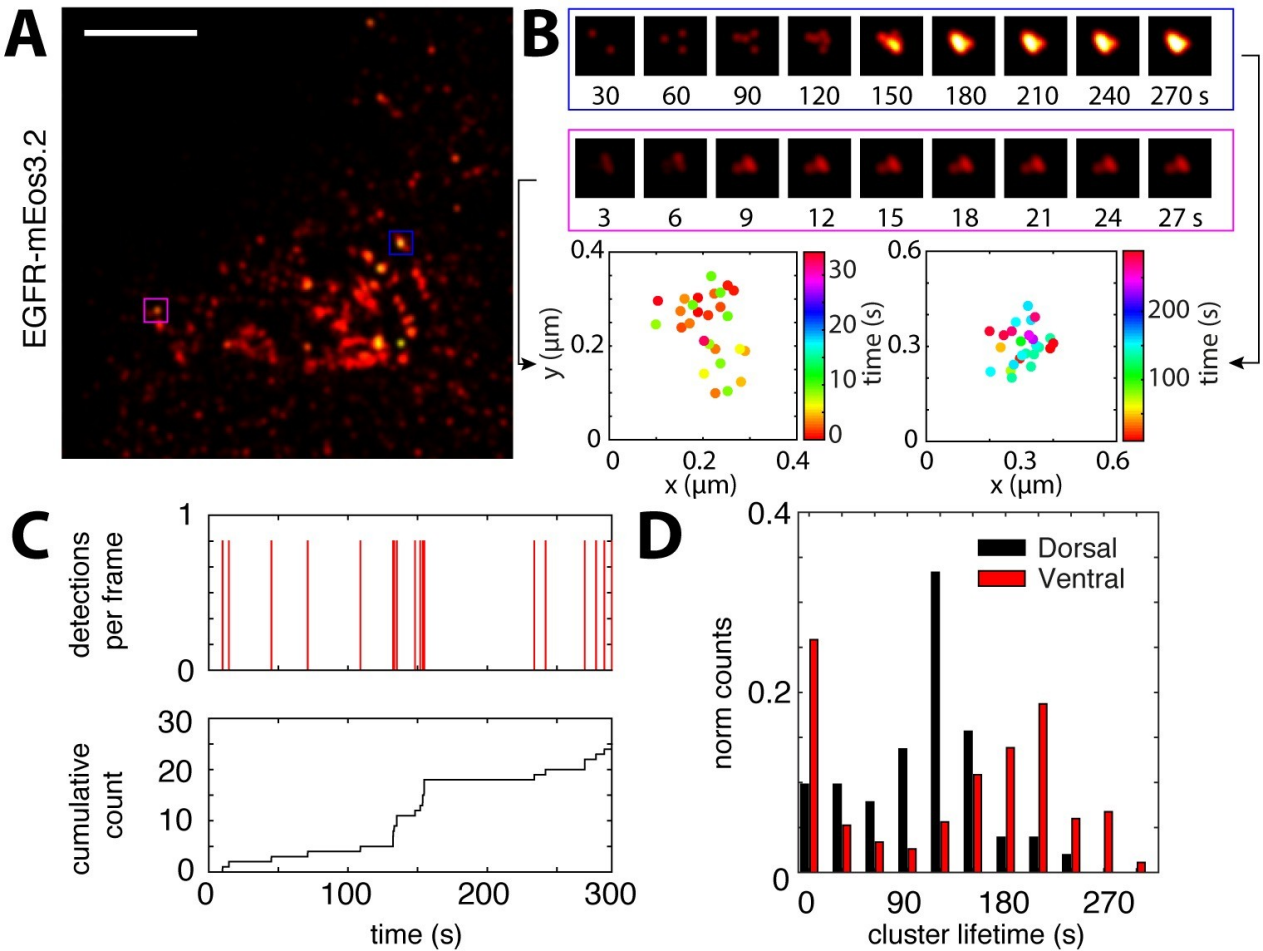
Live-cell super-resolution imaging reveals long-lived EGFR clusters in A549 cells. EGFR coupled to the photo-convertible protein mEos3 was expressed in A549 cells. Subsequent PALM imaging allows to study EGFR distribution in live cells at the single protein level. In the absence of any stimulus, we could detect nanodomains of EGFR within the dorsal and also the ventral plasma membrane (**A**). Scale bar: left panel, 1 μm. The image in **A** shows a maximum projected map of single molecule localizations recorded over a period of 10 min. **B** shows two cluster examples as a cumulative density distribution (upper panel) as well as XY scatter with the colorscale according to time at which the localization was detected (lower panel). While the projection of all localization allows to identify protein clusters, we can use the time information to further estimate the cluster lifetime. As shown in **C**, cumulative counting of individual localizations within a clustered region gives direct information of the minimum cluster lifetime. **D** shows the corresponding lifetime distribution of EGFR clusters recorded at the dorsal as well as the ventral side of the cell in the absence of any stimulus.

When an infecting IAV encounters an EGFR cluster, we would expect EGFR to become activated. Thus, we wanted to measure the cell’s EGFR response to IAV stimulation. Notably, in order to keep the signal at the plasma membrane, endocytosis was slowed down by stimulating the cells on ice. We used an antibody that recognizes a phosphorylated tyrosine (Y1068, pEGFR) previously shown to be involved in IAV-induced EGFR activation [9]. Interestingly, we found a fraction of pEGFR in nanodomains even under unstimulated conditions indicating pre- and/or self-activation. Following stimulation with either EGF or IAV, we observed an increased number of pEGFR clusters per area on the plasma membrane (Fig 5E). Similar to IAV-induced EGFR stimulation, the increase in local cluster density was sensitive to EGFR kinase inhibition (S14A and S14B Fig).

To better understand how IAV binding leads to EGFR activation, we examined the properties of individual EGFR clusters. It was previously hypothesized that IAV binding leads to a local concentration increase in EGFR proteins within plasma membrane clusters eventually leading to signal activation [9], an effect that was also observed before in BHK cells upon EGF stimulation [29]. To test this hypothesis, we labeled unstimulated, EGF- or IAV-stimulated cells using anti-EGFR antibodies. After EGF stimulation, we observed a decrease in the clustered molecule population as well as the number of clusters per area (both by on average 20%) (Fig 4F). Surprisingly, we did not find evidence for a significant redistribution of EGFR after IAV stimulation (Fig 4F). In addition, although we observed IAV colocalized with EGFR and pEGFR (S10 Fig), we could not detect an effect on the cluster size or the number of molecules per EGFR cluster (S14C Fig).

### EGFR forms long-lived nanodomains in living cells

Since IAV stimulation did not affect EGFR organization, we hypothesized that pre-formed nanoclusters might be involved in IAV-induced EGFR activation. While IAV engagement of pre-formed EGFR clusters was previously hypothesized [30], it remained unclear whether EGFR cluster lifetimes in the plasma membrane would be long enough to allow this type of interaction. To estimate the lifetime of EGFR clusters, we turned to live-cell microscopy experiments using A549 cells expressing EGFR tagged with the photo-convertible protein mEos3.2 [31]. We used an EGFR variant that was previously shown to be fully functional in a mammalian cell expression system [32]. Photoactivation of only a small subset of EGFR-mEos3.2 molecules allowed us to localize individual molecules which can then be tracked over consecutive frames, and renewed by further photoactivation (single-particle tracking, sptPALM) [33]. We performed sptPALM on the dorsal as well as the ventral plasma membrane of live A549 cells in the absence of EGF, resulting in high-density protein diffusion mapping (Fig 5). Calculation of the mean-squared displacement (MSD) as a function of the lag time 𝚫*t* allowed us to determine the instantaneous diffusion coefficient *D* along the trajectory. We found a broad range of diffusion coefficients ranging between 1 and 10^−3^ μm^2^/sec (S15 Fig), indicating that mobile and immobile protein fractions coexist in the plasma membrane. Indeed, after classifying trajectories based on their diffusion coefficient *D*, MSD vs. 𝚫*t* plots revealed a rather linear relationship for trajectories with *D* > 0.05 μm^2^/sec, while for trajectories with *D <* 0.05 μm^2^/sec the curve approached a maximum at large 𝚫t values (S15 Fig). This characteristic time dependence of the MSD vs. 𝚫*t* curve also indicates mobile freely diffusing proteins co-existing with confined or immobile EGFR proteins in the plasma membrane.

The same data was then used to construct a map of all detected localizations, which revealed a non-homogeneous distribution with proteins clustered into nanodomains (Fig 5A). Using our custom cluster identification analysis (as shown in Fig 2A–2C), we found EGFR clusters with diameters ranging between 30 and 300 nm, confirming the organization of EGFR observed in fixed cells (Fig 4A). In addition, and because the cells were alive, we could use the time-resolved single molecule detection to quantify the temporal stability of EGFR clusters (Fig 5B). Such an approach was used previously to quantify polymerase 2 clustering in live cells and is referred to as time-correlated PALM (tcPALM) [34]. We selected regions identified by density-based clustering to perform tcPALM (Fig 5B and 5C), and obtained a distribution of EGFR cluster lifetimes with an average of 140 seconds (Fig 5D, dorsal). Notably, this lifetime only provides a lower estimate since the cluster might have existed before starting the acquisition and might also be present after the last cluster molecule was photobleached.

## Discussion

**Fig 6:**
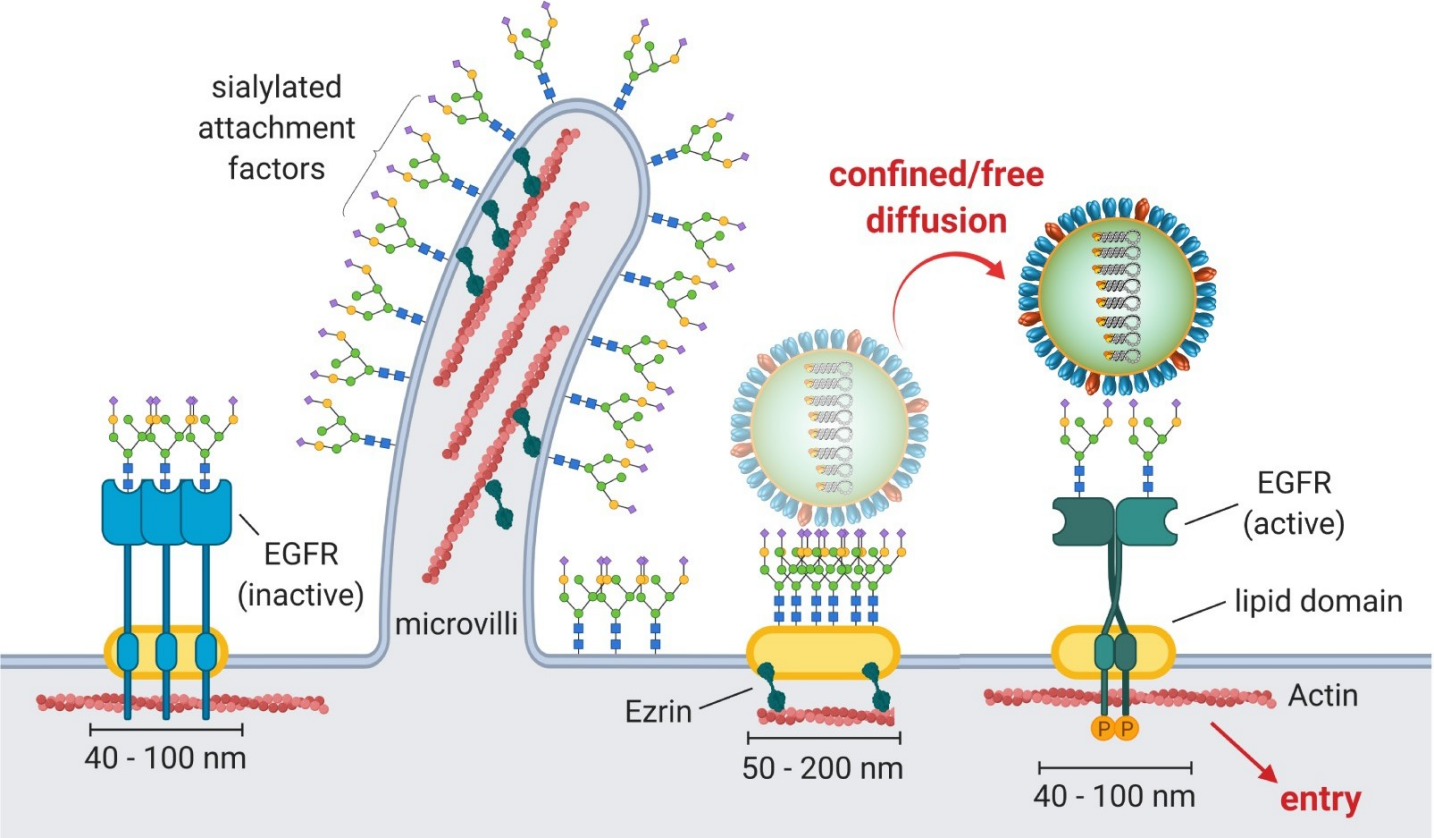
Model for IAV-mediated cell binding, receptor search and activation. Using quantitative STORM imaging, we could show that SA-conjugated IAV AF as well as one functional receptor, EGFR, form nanodomains in the plasma membrane of A549 cells. While dense AF nanodomains constitute an attractive multivalent binding platform, their diversity in local AF concentration suggests a variety of different residence times for which IAV would stay bound within these domains. Using single-virus tracking, we observed a mixed diffusive—confined motion, that could be simulated using our quantitative SA cluster information. These data suggest a receptor concentration-driven lateral search mechanism between SA enriched nanodomains. Eventually, since AF domains partly overlap with EGFR, IAV encounters a functional receptor that can be activated to signal cell entry. Our data further suggest that a stable preformed EGFR cluster population is activated during IAV stimulation, thereby possibly facilitating efficient signal transduction. EGFR clusters are stabilized by lipid rafts as well as cortical actin.

Understanding the initial phase of virus infection is crucial for the development of effective countermeasures such as adhesion inhibitors that catch viral particles before they can engage in the first virus-cell contact [35,36]. After binding sialylated AFs on the cell surface, IAV must find and activate a functional receptor to enter and infect its target cell. While these two steps are crucial for IAV infection, it remained largely speculative how the virus manages to form a multivalent cell contact while retaining flexibility to explore the plasma membrane and engage with a functional receptor. Here we used super-resolution microscopy to study the organization of virus-interacting molecules at the scale of the virus-cell interface. We found that glycan AFs and receptors are organized in nanoscale domains and provide a model for how cell binding can translate into receptor engagement and activation. Our findings are summarized schematically in Fig 6.

From the illustration it becomes clear that microvilli are a dominant topographical feature of the dorsal side of the cell. However, due to their diameter, they are not involved in virus endocytosis. We thus concentrated our efforts on analyzing the area between the microvilli and could identify a population of differently sized AF nanodomains. Our virus-binding simulations indicated that such a clustered organization of AFs is advantageous and positively correlates with the efficiency of virus binding (S7 Fig). A heterogeneous cluster organization might thereby broaden the binding capability of the cell surface and effectively provide the virus particle with a range of binding times to explore the cell surface as it was suggested before for Dendritic Cell-Specific Intercellular adhesion molecule-3-Grabbing Non-integrin (DC-SIGN) [37]. Such a binding mode could be particularly relevant for influenza viruses, which have a large morphological variability and a spike protein with strain-dependent affinity and specificity.

Single-virus tracking of fluorescently-labeled IAVs on and inside target cells [5–7] revealed that after forming the initial cell contact, IAV particles explore the plasma membrane for on average 6 minutes before endocytosis [6]. More recently, multicolor imaging of IAVs together with clathrin showed that this period is important for the induction of clathrin-dependent endocytosis [7]. We performed single-virus tracking under conditions of limited endocytosis to restrict virus motion to the plasma membrane. We mostly found trajectories where random diffusion alternates with periods of spatial confinement. The estimated sizes of the confined regions was consistent with the area found for AF nanodomains using STORM. This result is in agreement with our basic hypothesis that virus plasma membrane motion is largely influenced by the local organization and density of available AFs. Although we cannot exclude that IAV confinement is induced by other factors such as mechanical trapping, our results allow a model to be formulated of how the clustered organization of AFs controls virus-cell binding early during infection.

Having formed a stable interaction with its host cell, IAVs enter the cell by endocytosis. EGFR was shown to be activated during and promote IAV-cell entry [9], but how IAV engages with EGFR remained unclear. We found that EGFR localizes in clusters which colocalize with AF nanodomains. Regarding our results and those of others (reviewed recently in [38]), the current view of the plasma membrane suggests a compartmentalization into highly heterogeneous nanodomains with different origin and function. Colocalization at the nanoscale is thus measured as spatial proximity or co-clustering. The observed overlap between IAV AF and receptor clusters thus suggests that the lateral organization of AFs and EGFR underlies similar but not identical principles. Importantly the observed organization would indeed allow IAV to bind EGFR while moving in and between AFs domains (Fig 6).

In the canonical activation model, EGFR binds its substrate EGF, undergoes dimerization and subsequent autophosphorylation, thereby inducing a variety of signaling cascades [39]. However, as shown here on A549 cells, an additional level of higher oligomeric EGFR clusters has been shown across different cell types. Their reported diameter ranges from 50 nm [29] over 100–300 nm [26,30,40] up to near micrometer sizes [41], with molecule numbers between <10 [42] up to thousands [41]. Clustering was suggested to facilitate receptor activation which might play a role in tumor development [30]. Further, the EGFR cluster size was shown to respond to activation [29] suggesting a lateral molecule redistribution. With respect to the clustered EGFR organization, we hypothesized two scenarios in which, during activation, EGFR either (1) assembles into activated clusters or (2) pre-existing clusters become activated. Hence, we tested the organization of EGFR under (un)stimulated conditions. While we observed a reduction of the clustered molecule fraction as well as the number of clusters per area upon EGF stimulation, an effect that was observed previously for Erb2 [43], we did not detect a major redistribution in response to IAV attachment. This result speaks for the activation of pre-existing clusters and might also indicate a different activation mechanism. EGFR binds EGF with high affinity (nM), leading to fast EGFR activation (within 10 min [44]) and internalization. IAV binds EGFR using sialylated glycans, a low affinity process that requires some degree of multivalency. Our colocalization experiments showed that IAVs also bind multiple EGFR clusters (S10 Fig) indicating a different recruitment/activation mechanism. How IAV then eventually activates EGFR is still unclear and will be the objective of future investigation. What has been shown previously is that IAV-induced EGFR endocytosis is indeed slower compared to the EGF-induced activation, presumably due to the different mechanism of binding and activation [9]. Using STORM, we could not detect a change in cluster size and molecule composition following either stimulation. We hence conclude that intercluster spatial rearrangements below our resolution limit might eventually lead to cluster activation. Methods that are more sensitive to protein-protein distances below 20 nm such as FRET could be used to test this hypothesis.

Following our hypothesis of the activation of pre-formed EGFR clusters, we aimed to test if EGFR clusters exist long enough to allow their activation. We thus went on to probe the stability of EGFR nanoclusters by conducting live-cell sptPALM experiments. We found that the lifetime of EGFR nanodomains in both, the dorsal as well as the ventral side of the cell went up to 2 to 4 minutes. This time resembles the duration IAVs where shown to stay on the plasma membrane before onset of endocytosis (2–3 min [7]). While a long cluster lifetime would allow activation of pre-existing clusters, this observation raises the question for the stabilization of EGFR clusters. Hence, we tested the stability of EGFR clusters upon treatment with classical membrane domain-destabilizing conditions such as actin depolymerization (latrunculin A) and cholesterol extraction (methyl-β-cyclodextrin). As also observed before [29,30], our results suggest cluster destabilization following both perturbations indicated by an increased fraction of unclustered EGFR molecules. Interestingly, we found a stronger effect after cholesterol depletion, a condition that was previously shown to attenuate IAV replication [9]. Very long receptor cluster lifetimes were observed previously for class I major histocompatibility complex (MHC) molecules, that form actin-stabilized domains [45,46]. At this point, we can only speculate about the function of membrane receptor clusters [38,47]. It can be excitatory as shown for T-cell receptor [48] and linker for activation of T-cell (Lat) [49,50] as well as LFA-1 [51] or CD36 [52]. These nanodomains render the cell highly sensitive to small amounts of signaling molecules and due to the high local receptor concentration allow a fast signaling response [39]. Such a function seems likely for EGFR clusters involved in IAV cell entry observed in our study. But their function can also be inhibitory as shown for the B-cell receptor [53] or its negative co-receptor CD22 [54]. Receptor nanodomains could even engage in modulating the signaling output. As EGFR sits at the top of a broad array of signaling cascades, an asymmetric distribution of receptors could enable cells to rapidly respond and process different stimuli [39].

In summary, we identified a complex compartmentalization of the cellular plasma membrane in A549 cells with both of the primary IAV-binding molecules, sialylated AFs and functional receptors, organized in nanodomains. Such an organization was observed for other membrane proteins and might indicate a general organization principle of the plasma membrane (reviewed in [38]). We developed a functional model to link the lateral membrane organization to its role in virus infection (Fig 6). Quantitative super-resolution microscopy has provided a versatile toolbox to study the structure-function relationship of the plasma membrane. Our study provides a first step in understanding the nanophysiology of virus infection. By relating organization to function within the virus-cell interface, our goal is to better understand how viruses use cellular structures. How membrane compartmentalization can modulate IAV cell and receptor binding as shown here will be complemented with functional studies investigating the involved signal activation in the future.

## Materials and methods

### Ethics statement

Work with embryonated chicken eggs was conducted in the lab of Prof. Andreas Herrmann (Institute for Biology, Humbold-Universität zu Berlin, Germany) in accordance with European regulations and approved by the Berlin state authority, Landesamt für Gesundheit und Soziales. Influenza A (H3N2) X-31 was propagated in the allantoic cavities of 10-day old embryonated chicken eggs (Lohmann Tierzucht GmbH, Germany) as described previously [55].

### Cells and viruses

A549 cells (ATCC CCL-185) were kindly provided by Dr. Thorsten Wolff (Robert-Koch Institute Berlin, Germany). A549 cells were cultured in Dulbeccos Modified Eagles Medium (DMEM), supplemented with 10% fetal calf serum (FCS). The cells were passaged every 3–4 days. The cells were not polarized. We refer to the dorsal and ventral membrane as the upper cell surface as well as the part of the cell attached to the substrate. One day prior to the experiment, the cells were detached from the cell culture flask using 0.5% Trypsin/EDTA for about 10 min. The cells were diluted in fresh DMEM and 3*10^5^ cells were seeded on fibronectin-coated 25 mm round glass slides (Menzel, # 1.5). Influenza A (H3N2) X-31 was propagated in the allantoic cavities of 10-day old chicken eggs (Lohmann Tierzucht GmbH, Germany) as described previously [55]. Purified viruses were stored at -80°C. Virus aliquots were thawed on the day of the experiment and kept on ice until further use. All chemicals if not otherwise stated where purchased from Sigma-Aldrich. Cell culture media were purchased from Life Technologies. For the NA treatment, we use the Neuraminidase from *Clostridium Perfringens* (Sigma) which cleaves α2,6 and α2,3 linked SA.

### A549 cell infection

One day prior to the experiment, 3*10^5^ cells were seeded on fibronectin-coated 25 mm round glass slides. For infection experiments (S1 Fig), the cells where either incubated in serum-free medium for 30 min (control) or in DMEM supplemented with 100 ng/ml human EGF (R&D Systems) for 90 min to remove EGFR from the cell surface. Cells were infected with IAV X-31 (MOI ~1) in infection medium (DMEM, 0.2% bovine serum albumine (BSA)) for 30 min before the medium was changed and the cells were further incubated for 8 h in infection medium. The cells were washed in pre-warmed PBS and fixed in freshly prepared 4% PFA (Alpha Aesar) for 10 min at room temperature. We controlled that our fixation protocol could successfully immobilize plasma membrane molecules in A549 cells (S2 Fig, S16 Fig). After a 25 min fixation/blocking step in PBS supplemented with 0.2% Triton X-100 and 0.2% BSA, the cells were incubated with the primary antibody (anti influenza nucleoprotein (NP), Millipore) for 1 h. The cells were washed three times 10 min in PBS before further incubated with secondary antibodies (goat anti-mouse, Alexa 555 conjugate, Life Technologies) for 1 h. Finally, the cells were washed in PBS, stained with DAPI (0,2 μg/ml in PBS for 10 min) and mounted on standard microscope slides with Mowiol (Carl Roth). The slides where imaged using a Zeiss Axioplan epifluorescence microscope. Ten overview images (20 x magnification) were acquired for each condition and nuclear NP signal was quantified using Cellprofiler [56]. For the colocalization (S10 Fig) and cell stimulation experiments (Fig 4, S1A Fig, S14 Fig), the cells were infected at MOI = 100. For more details on the establishment of the experimental system, please see S1 Text.

### A549 cell transfection

One day prior to transfection, 2*10^5^ cells were seeded on fibronectin-coated 25 mm round glass slides. The cells were transfected with plasmids encoding EGFR-mEos3.2 using Lipofectamine 2000 (Life Technologies) according to the manufacturers guidelines. EGFR-mEos3.2 was cloned from EGFR-GFP, a gift from Alexander Sorkin (Addgene plasmid # 32751). Using our transfection protocol, we tested the EGFR clustering at different expression levels. We observed that the cluster density per area, but not the cluster size was dependent on the expression level (S16 Fig).

### Single-virus tracking

IAV H3N2/X-31 were incubated with 50 μM of the lipid dye DiD (Life Technologies) for 2h at RT. To remove the free dye, viruses were either pelleted (50.000 g for 5 min) or purified using a NAP5 size exclusion column (GE Healthcare). DiD-labelled viruses were diluted in infection medium (DMEM, 0.2% BSA) to a final concentration of 20 μg/ml (protein content) and viral aggregates were removed using a 0.2 μm sterile filter. Labeled viruses were added to A549 cells grown in 35 mm poly-L-lysine coated glass bottom petri dishes (MatTek Corp.) and allowed to attach on ice for 10 min. The cells were washed with PBS and overlaid with 2 ml pre-warmed, serum and phenol red-free DMEM supplemented with 100 mM Hepes. Unless otherwise stated, the cells were kept either at 4 or 37 deg throughout the experiment. For the perturbation experiments, the cells were pre-incubated in DMEM supplemented with 50 μM nocodazole (Sigma) or 40 μM dynasore (Sigma) for 30 min. The drugs were kept present throughout the experiment. Low temperature incubation was achieved using a custom build microscope temperature chamber. DiD was excited with 633 nm laser light, which was reflected on the sample by a 488/633 nm dichroic mirror. Emission light was collected using a 60x PlanApo VC oil-immersion objective (Nikon) and imaged onto an EMCCD camera (Andor iXon, Andor Technology). Images were recorded at 2 frames per second for 10 min. Image stacks were processed and the trajectories were build using ParticleTracker for ImageJ [57]. The trajectories were further analyzed using custom MatLab (Mathworks) scripts. To identify and characterize temporal particle confinement, we used the method developed by Simson *et al*. [22] implemented into our custom analysis pipeline. Briefly, the algorithm takes a segment of the trajectory and determines if the particle moved according to a given free diffusion coefficient (*D*_*free*_) within the segment, i.e. if the particles stays in a predicted region. This is translated into a confinement probability *I*_*conf*_. Since the identification depends on the length of the segment *S* [22], we optimized S using simulated trajectories resulting in S = 5 s for our analysis.

### Trajectory analysis and single particle tracking simulations

Random brownian particle trajectories were generated using the script package *msdanalzer* [58] incorporated into a custom MatLab routine. Single virus trajectories were analyzed as described above. Trajectory and sub-trajectory analysis was performed using *msdanalzer* to retrieve diffusion coefficients from mean square displacement (MSD, <r^2^>) vs. lag-time (Δt) plots. MSD vs. lag-time plots were fitted according to the type of motional behavior, which was either free diffusion (<r^2^> = 4D_free_Δt) or, for sub-trajectory analysis, confined (sub)diffusion (<r^2^> = <r^2^> (1-A_1_exp (−4A_2_D_conf_Δt/<r2>)). We found for IAV a mean free diffusion coefficient D_free_ = 0.041 μm^2^/s.

Using D_free_ as well as a time step (Δt = 0.5 s), the displacement of a freely diffusing particle follows a Gaussian distribution with standard deviation given by σ = sqrt(4D_free_Δt). Temporal confinement was introduced by generating a sub-trajectory using D_conf_. For the dwell time simulation, we generated random trajectories that run into a confinement region characterized by D_conf_ with a diameter of 50–300 nm according to the size of SNA clusters from STORM measurements (S1 Movie). Confined regions were identified using the confinement probability I_conf_ and the time the particle spends confined with I_conf_ > threshold was taken as the dwell time. D_conf_ was varied as shown in Fig 2.

### Preparation of labelled lectins and antibodies

Unconjugated *Sambuccus nigra* agglutinin (SNA, VectorLabs) or anti-EGFR antibodies (Sigma) were diluted to 0.6 mg/ml in 100 μl PBS (supplemented with 50 mM NaHCO_3_). AlexaFluor 647 NHS ester (Life Technologies) or Star Red NHS ester (Abberior) was added at a final concentration of (150 μM) and the solution was incubated for 30 min at room temperature. 100 μl PBS were added and the solution was applied to a NAP5 size exclusion column (GE Healthcare) pre-equilibrated with PBS. 300 μl fractions were collected in a 96-well plate and analyzed by ultraviolet—visible spectroscopy (Nanodrop2000, ThermoFisher). Peak protein fractions were collected and the degree of labelling calculated. The labelled lectin and antibody fractions were stored at 4°C until further use.

### SMLM sample preparation

One day prior to the experiment, 3*10^5^ A549 cells were seeded on fibronectin-coated 25 mm round glass slides. For SNA imaging, the cells were washed in pre-warmed PBS and fixed for 10 min in freshly prepared 4% paraformaldehyde (Alpha Aesar). The cells were blocked in blocking solution (5% BSA in PBS) and incubated in 50 μg/ml SNA diluted in blocking buffer for 30 min. The cells were washed 3 times in PBS and post-fixed in freshly prepared 4% paraformaldehyde for 10 min at RT. For EGFR labelling, the cells were washes, fixed and blocked as described above then incubated with anti-EGFR primary antibodies conjugated to Alexa 647. For two-color imaging, the cells were incubated with unconjugated primary anti-EGFR antibodies for 1h at RT. The cells were washed three times in PBS and further incubated with a solution of 5 μg/ml Alexa 750-conjugated secondary antibodies (goat anti-mouse, Life Technologies) and 5 μg/ml Alexa 647-conjugated SNA. The cells were washed 3 times in PBS and post-fixed in freshly prepared 4% paraformaldehyde for 10 min at RT. A549 cells in Fig 1B were labelled with 10 ng/ml SNA-Alexa647 and 1μg/ml Hoechst33342 (Life Technologies) in DMEM.

### SMLM microscopy

EGFR single- and two-color STORM imaging were performed using a recently developed flat-field epi illumination microscope [59]. Briefly, two lasers with wavelengths of 642 nm (2RU-VFL-P-2000-642-B1R, MPB Communications) and 750 nm (2RU-VFL-P-500-750-B1R, MPB Communications) were used to switch off fluorophores on the sample, while a 405 nm laser (OBIS, Coherent) controlled the return rate of the fluorophores to the fluorescence-emitting state. A custom dichroic (ZT405/561/642/750/850rpc, Chroma) reflected the laser light and transmitted fluorescence emission before and after passing through the objective (CFI60 PlanApo Lambda Å~60/NA 1.4, Nikon). After passing the respective filter (ET700/75M, Chroma or ET810/90m, Chroma), emitted light from the sample was imaged onto the sCMOS camera (Zyla 4.2, Andor). Axial sample position was controlled using the pgFocus open hardware autofocus module (http://big.umassmed.edu/wiki/index.php/PgFocus). Typically, 20,000 frames at 10 ms exposure time were recorded using Micromanager[60]. Imaging was performed using an optimized STORM buffer as described previously[61]. Image stacks were analyzed using a custom CMOS-adapted analysis routine[62]. Lateral sample drift was corrected using either image correlation (Thunderstorm[63]) or gold fiducial markers (B-Store, https://github.com/kmdouglass/bstore). Two-color datasets were analyzed using LAMA[64]. Random datasets for CBC analysis were generated in MatLab.

SNA single color imaging was performed on a modified Olympus IX71 inverted microscope. A 641 nm laser (Coherent, CUBE 640-100C) and a 405 nm laser (Coherent, CUBE 405-100C) was reflected by a multiband dichroic (89100 bs, Chroma) on the back aperture of a 100x 1.3 NA oil objective (Olympus, UplanFL) mounted on a piezo objective scanner (P-725 PIFOC, Physik Instrumente). The collected fluorescence was filtered using a band-pass emission filter (ET700/75, Chroma) and imaged onto an EMCCD camera (IxonEM+, Andor) with a 100 nm pixel size and using the conventional CCD amplifier at a frame rate of 25 fps. Laser intensity on the sample measured after the objective was 2–4 kW/cm^2^. 20,000 frames at 30 ms exposure time were recorded using Micromanager[60]. Image stacks were analyzed using ThunderStorm[63]. Lateral sample drift was corrected using either image correlation (Thunderstorm[63]) or gold fiducial markers (PeakSelektor, IDL, courtesy of Harald Hess).

PALM imaging was performed on a Zeiss Axio Observer D1 inverted microscope, equipped with a 100x, 1.49 NA objective (Zeiss). Activation and excitation lasers with wavelengths 405 nm (Coherent cube) and 561 nm (Crystal laser) illuminated the sample in total internal fluorescence (TIRF) mode. We used a four color dichroic 89100bs (Chroma), fluorescence emission was filtered with an emission filter ET605/70 (Chroma) and detected with an electron-multiplying CCD camera (iXon+, Andor Technology) with a resulting pixel size of 160nm. For each region of interest, typically 10000 images of a 41x41 μm2 area were collected with an exposure time of 30 ms. Photoactivatable proteins were activated with 405 nm laser intensity < 0.5 W/cm^2^, chosen to maintain a sparse population of activated molecules for localization, and excited with 561 nm laser intensity of ~1 kW/cm^2^. Image stacks were analyzed using ThunderStorm[63].

### STED microscopy

Live cell STED measurements were done with Abberior STED microscope (Abberior Instruments, Germany) as previously described in [65,66]. The microscope is equipped with a titanium-sapphire STED laser (MaiTai HP, Spectra-Newport). The labelled Abberior Star Red- labelled SNA was excited using 640 nm pulsed diode laser (Picoquant, Germany) with an average excitation power of 5–10 μW at the objective (UPlanSApo 100x/1.4 oil, Olympus). Depletion was achieved using tunable pulsed laser at 780 nm. The microscope was operated using Abberior’s Imspector software. Fixed-cell STED measurements (S3 Fig) were performed on a Leica SP8 STED microscope. The microscope was equipped with a HC PL APO 100x objective, a white light laser for excitation and a 592 nm depletion laser.

### Cluster analysis

For the cluster analysis, we used the algorithm density-based spatial clustering applications with noise (DBSCAN)[67], which was embedded into our custom analysis MatLab routine. DBSCAN only needs two input parameters, *Eps* and *k*. It then counts for each localization, how many neighbor localization are within a circle of radius *Eps*. If the localization has *k* neighbors within *Eps*, it is classified as part of a cluster. If it does not have enough neighbors within *Eps*, but is itself a neighbor of a cluster localization, it is classified as an edge point. All remaining localization are classified as unclustered. In order to analyze the very dense and heterogeneous localization maps we obtained from SNA imaging, we performed two consecutive DBSCAN runs with different parameters for *Eps* and *k*. Only this allowed us to account for all visually visible clusters. Clustered and edge points are then combined and handed over to the single cluster analysis part of the analysis routine. For each cluster, we examined a set of parameters such as area and mean diameter as well as the number of localizations per cluster. We further analyzed the localization density distribution per cluster by performing a nearest-neighbor search using a search radius of 20 nm. All localization processing was performed using custom written MatLab (MathWorks) scripts.

### Single molecule calibration

In order to measure the localization precision of our system and calibrate the grouping parameters, we performed STORM imaging on isolated dye molecules. 25 mm round glass slides (Menzel, # 1.5) were plasma cleaned for 10 min and coated with poly-L-lysine solution (100 μg/ml in ddH_2_O) for 1 h at room temperature. After washing in ddH_2_O, the slides were dried and incubated with 10–50 pM dye-conjugated SNA or anti-EGFR antibodies respectively. After 15 min, the slides were washed once and then imaged under experimental conditions. Localization maps were filtered and drift-corrected using gold fiducials. Individual localizations were first grouped with *gap = 0* and search radius 30 nm, to merge individual blink events, then grouped again with a gap time equal to the total acquisition time (15k frames). This allows to quantify the spread of localizations along x and y *𝜟(x*,*y)* (i.e. the localization precision σ) as well as the time between individual blink events (i.e. the dark time *t*_*D*_). All localization processing was performed using custom written MatLab (MathWorks) scripts.

### Virus binding simulations

We simulated a flat patch of cell surface (1x1 μm^2^) including *n* attachment factor molecules at random positions. For the analysis of the degree of clustering (S7B Fig), the simulated molecules were gradually shifted into clusters, while *n* was kept constant. To analyze the impact of the cluster size (i.e. the number of receptors per cluster), we simulated a constant concentration of attachment factor molecules and added receptor clusters at the indicated size (S7A Fig). A virus attempting to attach was simulated as s two-dimensional projection of a spherical 100 nm virus particle. The virus center was randomly placed onto the simulated surface and the number of attachment factor molecules within a radius of 50 nm was counted. More than 10 molecules were counted as successful binding. Matlab scripts to run the simulation are available at GitHub (https://github.com/christian-7/Virus_Binding_Simulation).

## Supporting information

S1 Text Establishment of the experimental system. (DOCX)

S1 Fig Establishment of the experimental system. We first tested if our experimental system (virus and cells) is suitable for the intended experiments. Virus binding was tested at high MOI after low-temperature adsorption followed by immunostaining using anti-H3N2 antiserum. We found that our IAV strain could efficiently bind to A549 cells at high MOI (~100) (**A**). Individual cells are highlighted (dashed lines). IAV infection was performed at lower MOI (~1) to achieve a high contrast infection and avoid overinfection. Following infection, the cells were incubated for 8h, then fixed and immunostained using anti-NP antibodies. The infection was quantified as nuclear NP accumulation and analyzed using CellProfiler [56] (NP/DAPI ratio) (**B**). We found that our influenza A H3N2/X31 virus could efficiently infect A549 cells at a titer of 8.3*10^5^ focus forming units (FFU) per ml. A549 cells were either treated with 100 ng/ml EGF for 30 min to reduce the concentration of available cell surface EGF receptors or 0.01U/ml neuraminidase for 3h at 37°C. Cells were infected with influenza A/X31 (MOI ~ 1) for 8h then fixed and immunolabelled for newly produced viral nucleoprotein (NP) (**D**). The cell nuclei were counterstained with DAPI. Nuclear NP signal was quantified using automated image analysis with CellProfiler (**E, F**).
(PDF)

S2 Fig EGFR is immobilized after chemical fixation. A549 cells were transfected with plasmids encoding EGFR-mEos3.2 24h before imaging. On the day of the experiment, the DMEM growth medium was exchanged for Leibovitz medium and the cells were transferred into the microscopes sample holder and imaged using TIRF illumination for 10 min per field of view. Individual EGFR proteins could be localized and tracked over several frames. **A** shows a rendering of all localizations from a single field of view. **B** shows the trajectories of three EGFR molecules around a nanocluster (boxed area in **A**). We detected an immobile protein fraction of about 45%. Following PFA fixation, the EGFR mobility was strongly reduced leading to smaller clusters (**C,** rendered image; **D**, trajectories in boxed area) and an immobile protein fraction of >95% (**E**). To relate the amount of immobilization, we also tracked localizations stemming from gold fiducials immobilized on the glass slide (Bkgd in **E**).
(PDF)

S3 Fig Nanoscale glycan organization in A549 cells. A549 cells were fixed, labelled using SNA and imaged with STED. We found a similar glycan organization of small clusters as well as protruding microvilli as visualized with STORM (**A**). A similar glycan organization was observed using SNA on MDCK cells (**B**). We also labelled A549 cells with wheat germ agglutinin (WGA), which unlike SNA, less specifically labels all sialylated glycans (**C**). Using WGA, we could reproduce the nanocluster compartmentalization of the cell surface as well as protruding hollow microvilli (arrow heads in **C**, right panel).
(PDF)

S4 Fig Nanoscale Ezrin organization in A549 cells. (**A**) A549 cells were immunolabelled for the actin-binding protein Ezrin, which was shown to be enriched in microvilli [18]. The cells were imaged using STORM. Microvilli are clearly distinguishable and resemble the large cluster population as observed on SNA-labelled cells and in scanning electron microscopy (SEM, inset). Scale bars: left panel: 2 μm, right panel: 500 nm, inset: 200nm. (**B**) Ezrin localization maps can then be used to set a threshold for the clusters size obtained from DBSCAN clustering to specifically analyze the non-microvilli cluster population in SNA localization maps (Fig 2).
(PDF)

S5 Fig Experimentally obtained localization precision σ for Alexa 647 and Alexa 750. Glass slides were washed, plasma cleaned and coated with Poly-L-lysine (0.01% in water) for 1h. Conjugated antibodies were diluted in PBS to a final concentration of ~10 nM and adsorbed to the coated glass slides. Individual molecules were imaged under experimental conditions. Localizations originating from single Alexa 647 (**A**) and Alexa 750 (**B**) molecules were aligned to allow the estimation of the average localization precision: σ_x,y_ A647 = 12 nm and σ_x,y_ A750 = 21 nm.
(PDF)

S6 Fig Localization precision partly mimics local concentration gradient. To test if the localization precision accounts for the gradient in localization density we observed in AF clusters (Fig 3), we simulated clusters of random localizations (**A**) using cluster size data taken from our experimental STORM measurements (i.e. radius *r*, number of localizations *n*, localization precision *σ*). The local density was then determined using a nearest neighbor search within a radius of 3*σ*. We indeed observed that the simulated clusters exhibit an up to about 8-fold local enrichment (see one example in **A**-**C**). We then simulated clusters following the full distribution of experimental data (i.e. radius *r*, number of localizations *n*). Comparing with the density gradient observed in our experimental data (**D** andFig 3), we find that both distributions are well separated and that the described effect only accounts for density changes < 8-fold.
(PDF)

S7 Fig A clustered AF organization increases IAV binding probability. To estimate the effect of AF clustering on the efficiency of a virus to bind the target cell, we simulated two scenarios in a 1x1 μm membrane area, (**A**) a varying cluster size and (**B**) a varying degree of clustering. For **A**, we simulated a constant lateral concentration of AF (black) and added AF clusters (blue) at increasing size. In **B**, we keep the total amount of AF constant and gradually shift molecules into clusters. In both cases, an approaching virus was simulated as a 2D projection of a small spherical IAV particle (contact area as red circles in **A** and **B**). A binding attempt was counted as successful if at least 10 AF molecules were found inside the contact area. **C** and **D** show the simulation result plotted as the binding probability out of 1000 simulations against the respective tested cluster parameter.
(PDF)

S8 Fig Confinement probability can robustly identify transient immobilization in IAV trajectories. To test the precision of the confinement probability, we simulated random trajectories that did not or did contain a confined region and calculated the confinement probability I_conf_ for each trajectory. The confined region was chosen according to experimental data for the radius (r = 50–300 nm) as well as D_conf_ (Figs 2 and 3). To identify a confined region, we had to set a threshold of I_conf_, which we set to 5 to allow high sensitivity. Our simulations (100 trajectories) show that at this threshold we have a chance of false identification (false positives) of ~10%, which drops to 0% at I_conf_ > 8 (**A**). For the simulations that did contain a confinement (example in **B**), we found an average detection efficiency (true positives) of 90% across the entire simulation space (900 trajectories, color code in **C**).
(PDF)

S9 Fig IAV single-virus tracking on A549 cells. Single virus tracking on live A549 cells revealed four main types of virus movement: (**A**) three-stage movement, (**B**) confined, (**C**) mixed, (**D**) drift. The fraction of all modes of movement was analyzed at the indicated conditions (**E**).
(PDF)

S10 Fig IAVs colocalize with EGFR and pEGFR on A549 cells. A549 cells were infected with IAV X31 (MOI = 100) at low temperature for 15 min, then fixed and immunostained using anti-H3N2 antiserum together with either anti-EGFR or anti-pEGFR (Y1068) antibodies. We analyzed images takes in widefield epi illumination (**A**) as well as two-color STORM (**B**). Using widefield epi illumination, we observed bright IAV spots that co-localize with EGFR clusters and pEGFR signal (arrow heads). Scale bar = 2 μm. We quantified this colocalization and found that in both cases about 20% of IAVs colocalize with EGFR and pEGFR respectively. In the two-color STORM reconstructions, we could also observe IAVs that colocalize with EGFR or pEGFR. More specifically, due to the increased resolution, we could discriminate fully overlapping colocalization (**B**, arrow heads) as well as IAVs that associate with more than one (p)EGFR cluster (**B**, asterisk). Shown are overview images (left) as well as four magnified regions (right) for each overview.
(PDF)

S11 Fig EGFR-kinase activity is required for IAV-mediated EGFR activation and infection. A549 cells were infected with IAV X31 (MOI = 100) at 4°C for 15 min upon pre-treatment with 10 μM Gefitinib for 1h min at 37°C. The cells were fixed and immunostained using pEGFR (Y1068) antibodies to label activated EGFR (**A**) and nuclear DNA was labelled with DAPI. The cytosolic pEGFR signal was analyzed using ImageJ (**B**). To probe the infection efficiency, A549 cells were infected with IAV X31 (MOI = 1) for 8h upon pre-treatment with 10 μM Gefitinib for 1h min at 37°C. The cells were fixed and immunostained using anti-NP antibodies (**A**) and nuclear DNA was labelled with DAPI (**C**). The infected was quantified as nuclear NP/DAPI signal as analyzed with CellProfiler (**D**). EGFR nanoclusters are sensitive to actin- or lipid domain destabilization (**E**). A549 cells were incubated with latrunculin A (1 μM) or methyl-b-cyclodextrin (MCD) (40 μg/ml) for 1 h at 37°C, then fixed and stained using anti-EGFR antibodies.
(TIF)

S12 Fig Molecule blinking correction for EGFR data. To estimate the number of emitting molecules from a STORM dataset and to avoid false clustering of individual molecules, we merged multiple localizations originating from the same molecule into a single localization. The merging procedure requires a gap distance as well as a gap time, within which localizations will be counted as originating from the same molecule. To calibrate these values, we imaged isolated labelled anti-EGFR antibodies under experimental conditions. Localizations originating from a single molecule could be grouped to determine their lateral spread (**A**) as well as the dark time between individual bursts (**B**). To ensure a high certainty of merging, the dark time cut-off was determined by the 99% quantile to 18 s. Using the experimentally determined spread of localization (**A**, 35 nm) and the dark-time cut off, localization bursts from the same molecule can now be combined into a single position. While each molecule is counted multiple times due to molecule blinking (uncorrected, **C**), merging allows a more precise estimate of the molecule numbers while avoiding false clustering (corrected, **D**).
(PDF)

S13 Fig Experimental positive control for two-color STORM colocalization analysis. We used two differently labelled versions of SNA as an experimental colocalization positive control. This served us also as a nominator to better evaluate the degree of colocalization of our test molecule pair SNA/EGFR. A549 cells were labelled with two SNA variants, conjugated to Alexa 647 as well Alexa 555 (**A**). Both localization datasets were analyzed using CBC resulting in a colocalization value C_A_ associated to each individual localization. A histogram of C_A_ for one channel is shown in **B**. We set the threshold to 0.3, above which localizations were counted as colocalized. **C** shows one SNA dataset color coded according to C_A_. **D** shows all localizations from the same dataset with C_A_ > 0.3.
(PNG)

S14 Fig EGFR cluster size and molecule number do not change after IAV or EGF stimulation. EGFR cluster activation can be inhibited by the EGFR kinase inhibitor Gefitinib (**A, B**). A549 cells were infected with IAV X31 (MOI = 100) at 4°C for 15 min upon pre-treatment with 10 μM Gefitinib for 1h min at 37°C. The cells were fixed and immunostained using pEGFR (Y1068) antibodies to label activated EGFR. The cells were imaged using STORM and pEGFR cluster identified using DBSCAN. Following stimulation with EGF or IAV, we detected an increase in the number of pEGFR cluster per area (as also shown in Fig 5). This effect could be inhibited by Gefitinib treatment (**A, B**). A549 were treated with infection medium (control) or infection medium containing IAV (MOI = 100) or 100 ng/ml EGF for 15 min at 4°C. The cells were fixed and immunostained using anti-EGFR antibodies. Upon either stimulation, we could not detect a change in the size of the EGFR clusters or the amount of molecules per cluster (**C**).
(PDF)

S15 Fig Single molecule diffusion coefficients from sptPALM of EGFR-mEos3 in A549 cells. A549 cells were transiently transfected with EGFR-mEos3. sptPALM imaging and analysis of single molecule trajectories revealed a wide range of diffusion coefficients. The left panels in **A** and **B** show the distribution of diffusion coefficients from sptPALM obtained at the dorsal (**A**) and the ventral plasma membrane (**B**). Molecules were classified as mobile (D>0.5 μm2/s) or immobile (D<0.5 μm2/s) respectively. Calculated MDS plots (**A** and **B**, right panels) for both classes exhibit a rather linear dependence for the mobile fraction, while the curve saturates with increasing lag time for the immobile fraction, the latter indicating spatial confinement.
(PDF)

S16 Fig EGFR expression level does not correlate with the EGFR cluster size but with the number of clusters per area. A549 cells were transfected with plasmids for EGFR-mEos3.2 24h before the imaging experiments. On the day of the experiment, the cells were fixed and and imaged. In order to assess the expression level, we took a snapshot of the pre-converted green mEos version before photoconversion and PALM acquisition. We performed the DBSCAN cluster analysis for different cells at various expression levels (**A**). We observed that the number of clusters per area increased at higher expression level, while the cluster radius was not affected (**B**).
(PDF)

S1 Movie Evolution of the confinement probability I_conf_ as well as the particles distance from the origin for a simulated virus trajectory. The particle moves according to D_free_ until it encounters an AF cluster (top panel, red circles). Due to the higher concentration of AF, the particles diffusion is slowed down to D_conf_ and the particle is confined. Particle confinement is detected by an increase of the confinement probability (middle panel) as well as a plateau in the distance from origin plot (lower panel).
(AVI)
