# Supplementary material for: Influenza A viruses use multivalent sialic acid clusters for cell binding and receptor activation
Source: PLoS Pathog. 2020 Jul 8;16(7):e1008656. doi: 10.1371/journal.ppat.1008656 (PMC7371231; doi:10.1371/journal.ppat.1008656)
Supplement: S1 Text — (DOCX) [file ppat.1008656.s001.docx]

**Establishment of the experimental system**

The goal of this study is to describe the nanoscale structural organization of IAV AFs and functional receptors to develop a model for the initial phase of virus-cell binding and receptor activation. We used the lab-adapted IAV strain H3N2/X31 together with A549 human lung epithelial cells. We have previously tested the glycan specificity of our IAV strain and found it to recognize almost exclusively human type (α-2,6 conjugated) sialylated glycans [1]. We thus concluded that *Sambuccus Nigra Agglutinin* (SNA), a plant lectin that specifically recognizes α-2,6 conjugated sialylated glycans [2] is an adequate probe to sample the AF organization within our experimental system.

We then tested if our IAV strain efficiently binds and infects A549 cells. The goal of these experiments was to establish an MOI used for 1) infection experiments as wells as 2) EGFR perturbation experiments, where the latter typically requires a much higher MOI [3]. For these experiments we used two preparations of viruses, 1) cell supernatant from infected cells as well as 2) concentrated viruses following ultracentrifugation. Virus binding was tested at high MOI after low-temperature adsorption followed by immunostaining using anti-H3N2 antiserum. We found that our IAV strain could efficiently bind to A549 cells at high MOI (~100) (**S1A Fig**). IAV infection was performed at lower MOI (~1) to achieve a high contrast infection avoiding overinfection. Following infection, the cells were incubated for 8h, then fixed and immunostained using anti-NP antibodies. The infection was quantified as nuclear NP accumulation and analyzed using CellProfiler [4] (NP/DAPI ratio) (**S1B Fig**). The same procedure was used to assess the viral titer of our IAV sample (resulting in 8.3*10^5^ FFU/ml) (**S1C Fig**). The established procedures and MOIs were used for all other experiments presented in the manuscript.

It was shown previously that influenza A virus (IAV) activates and uses EGFR to trigger endocytosis and enter into mammalian host cells [3]. Specifically, this was shown for different virus strains including H1N1/PR8, H7N1/FPV and H3N2/X31 [3,5]. To test if IAV H3N2/X31 in our hands entered cells in an EGFR-mediated way, we stimulated human A549 cells with 100 ng/ml EGF, causing EGFR internalization and thereby removal from the cell surface [3]. We found that successful virus infection, as detected by viral nucleoprotein production, was decreased by 40 % compared to the control. As expected, the effect of complete removal of the primary IAV AF using sialidase using neuraminidase (NA) treatment was much stronger and reduced the amount of infected cells by 80 % (**S1D-F Fig**). As a second independent test we treated A549 cells with Gefitinib, a specific EGFR kinase inhibitor. We found that 10 µM Gefitinib reduced the EGF and IAV-mediated activation of EGFR as well as the infection efficiency by about 50 % (**S2A Fig**).

**References**

1. Kastner M, Karner A, Zhu R, Huang Q, Zhang D, Liu J, et al. Relevance of host cell surface glycan structure for cell specificity of influenza A virus. bioRxiv. 2017; Available: http://biorxiv.org/content/early/2017/10/14/203349.abstract

2. Shibuya N, Goldstein IJ, Broekaert WF, Nsimba-Lubaki M, Peeters B, Peumans WJ. The elderberry (Sambucus nigra L.) bark lectin recognizes the Neu5Ac(alpha 2-6)Gal/GalNAc sequence. J Biol Chem. 1987;262: 1596–1601.

3. Eierhoff T, Hrincius ER, Rescher U, Ludwig S, Ehrhardt C. The epidermal growth factor receptor (EGFR) promotes uptake of influenza A viruses (IAV) into host cells. PLoS Pathog. 2010;6: e1001099. doi:10.1371/journal.ppat.1001099

4. Carpenter AE, Jones TR, Lamprecht MR, Clarke C, Kang I, Friman O, et al. CellProfiler: image analysis software for identifying and quantifying cell phenotypes. Genome Biol. BioMed Central; 2006;7: R100. doi:10.1186/gb-2006-7-10-r100

5. Zhu L, Ly H, Liang Y. PLC-γ1 signaling plays a subtype-specific role in postbinding cell entry of influenza A virus. J Virol. 2014;88: 417–24. doi:10.1128/JVI.02591-13
