# Supplementary figures and images for: Influenza A viruses use multivalent sialic acid clusters for cell binding and receptor activation
Source: PLoS Pathog. 2020 Jul 8;16(7):e1008656. doi: 10.1371/journal.ppat.1008656 (PMC7371231; doi:10.1371/journal.ppat.1008656)

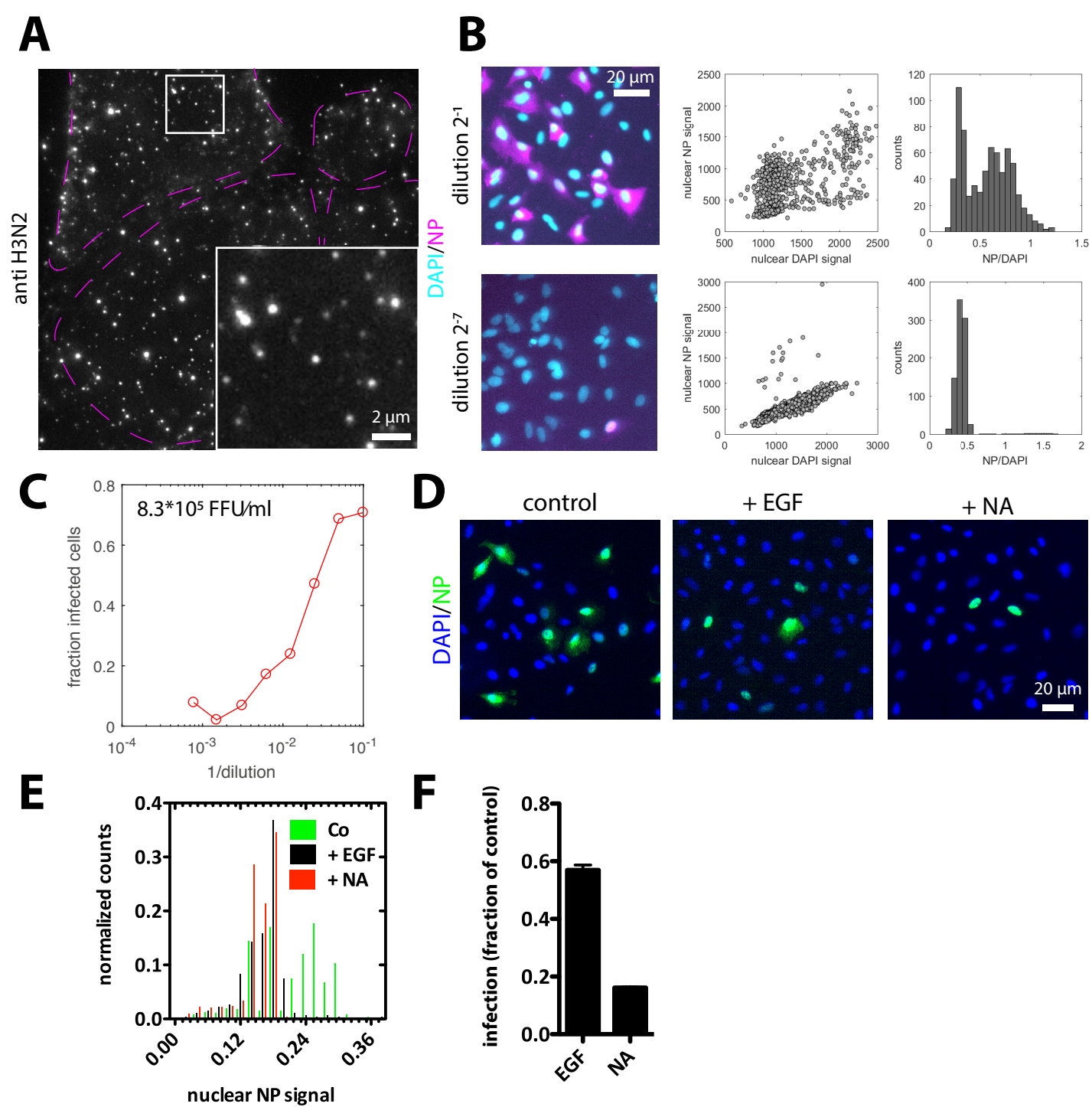

Supplement: S1 Fig — We first tested if our experimental system (virus and cells) is suitable for the intended experiments. Virus binding was tested at high MOI after low-temperature adsorption followed by immunostaining using anti-H3N2 antiserum. We found that our IAV strain could efficiently bind to A549 cells at high MOI (~100) (A). Individual cells are highlighted (dashed lines). IAV infection was performed at lower MOI (~1) to achieve a high contrast infection and avoid overinfection. Following infection, the cells were incubated for 8h, then fixed and immunostained using anti-NP antibodies. The infection was quantified as nuclear NP accumulation and analyzed using CellProfiler [56] (NP/DAPI ratio) (B). We found that our influenza A H3N2/X31 virus could efficiently infect A549 cells at a titer of 8.3*105 focus forming units (FFU) per ml. A549 cells were either treated with 100 ng/ml EGF for 30 min to reduce the concentration of available cell surface EGF receptors or 0.01U/ml neuraminidase for 3h at 37°C. Cells were infected with influenza A/X31 (MOI ~ 1) for 8h then fixed and immunolabelled for newly produced viral nucleoprotein (NP) (D). The cell nuclei were counterstained with DAPI. Nuclear NP signal was quantified using automated image analysis with CellProfiler (E, F). (PDF) [file ppat.1008656.s002.pdf]

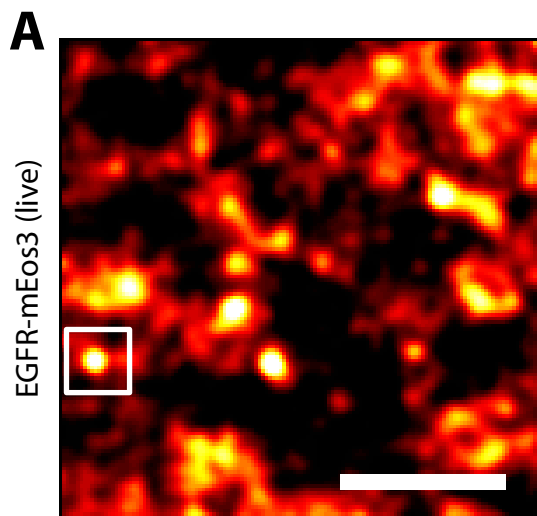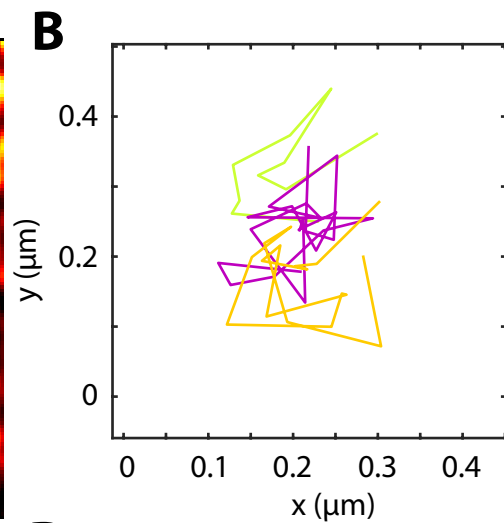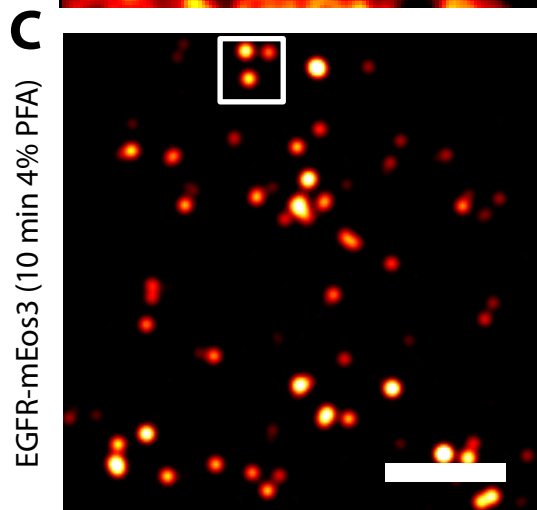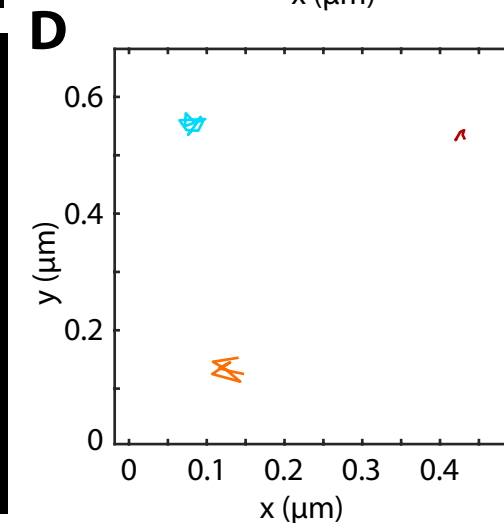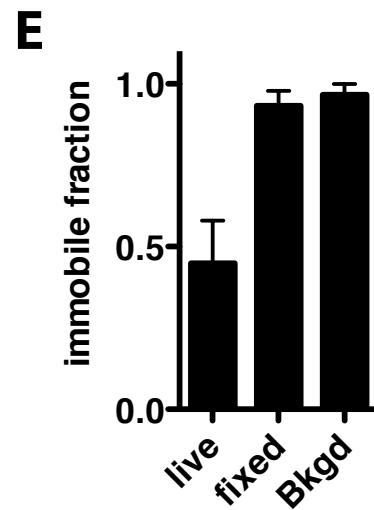

Supplement: S2 Fig — A549 cells were transfected with plasmids encoding EGFR-mEos3.2 24h before imaging. On the day of the experiment, the DMEM growth medium was exchanged for Leibovitz medium and the cells were transferred into the microscopes sample holder and imaged using TIRF illumination for 10 min per field of view. Individual EGFR proteins could be localized and tracked over several frames. A shows a rendering of all localizations from a single field of view. B shows the trajectories of three EGFR molecules around a nanocluster (boxed area in A). We detected an immobile protein fraction of about 45%. Following PFA fixation, the EGFR mobility was strongly reduced leading to smaller clusters (C, rendered image; D, trajectories in boxed area) and an immobile protein fraction of >95% (E). To relate the amount of immobilization, we also tracked localizations stemming from gold fiducials immobilized on the glass slide (Bkgd in E). (PDF) [file ppat.1008656.s003.pdf]

**A**      confocal      STED

SNA

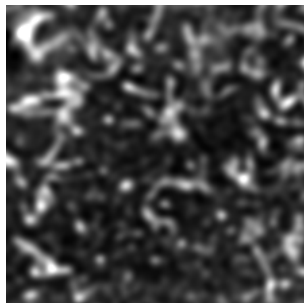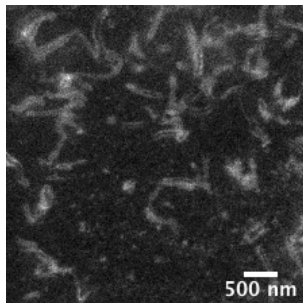

**B**

SNA, MDCK cells

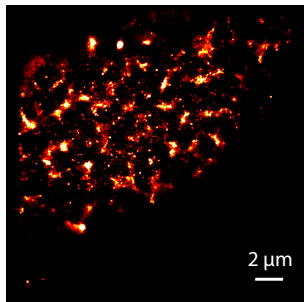

**C**

WGA, A549 cells

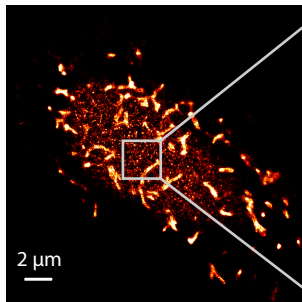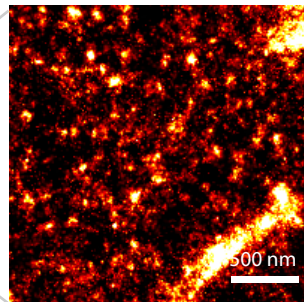

Supplement: S3 Fig — A549 cells were fixed, labelled using SNA and imaged with STED. We found a similar glycan organization of small clusters as well as protruding microvilli as visualized with STORM (A). A similar glycan organization was observed using SNA on MDCK cells (B). We also labelled A549 cells with wheat germ agglutinin (WGA), which unlike SNA, less specifically labels all sialylated glycans (C). Using WGA, we could reproduce the nanocluster compartmentalization of the cell surface as well as protruding hollow microvilli (arrow heads in C, right panel). (PDF) [file ppat.1008656.s004.pdf]

*STORM*

**A**

*Ezrin*

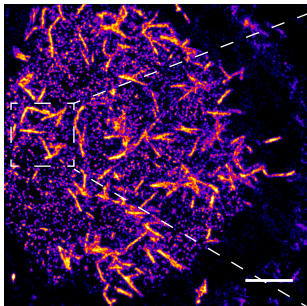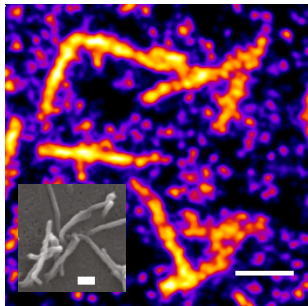

**B**

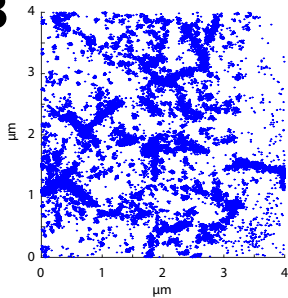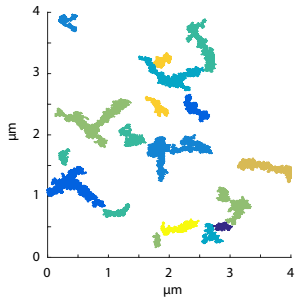

Supplement: S4 Fig — (A) A549 cells were immunolabelled for the actin-binding protein Ezrin, which was shown to be enriched in microvilli [18]. The cells were imaged using STORM. Microvilli are clearly distinguishable and resemble the large cluster population as observed on SNA-labelled cells and in scanning electron microscopy (SEM, inset). Scale bars: left panel: 2 μm, right panel: 500 nm, inset: 200nm. (B) Ezrin localization maps can then be used to set a threshold for the clusters size obtained from DBSCAN clustering to specifically analyze the non-microvilli cluster population in SNA localization maps (Fig 2). (PDF) [file ppat.1008656.s005.pdf]

**A**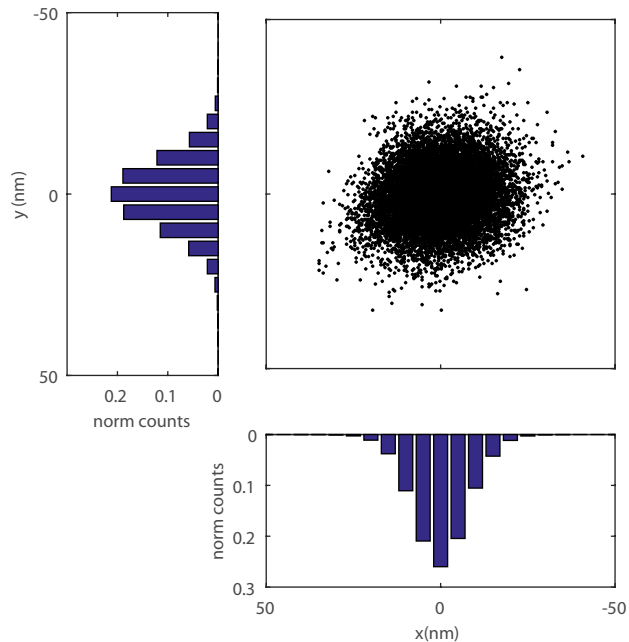**B**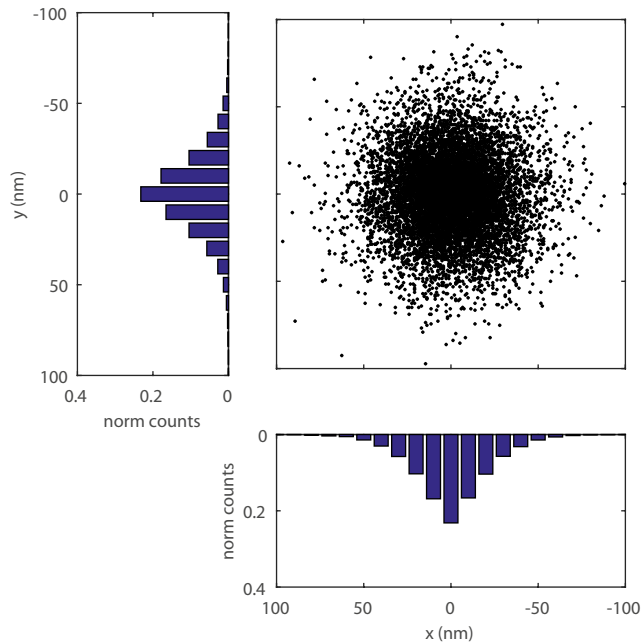

Supplement: S5 Fig — Glass slides were washed, plasma cleaned and coated with Poly-L-lysine (0.01% in water) for 1h. Conjugated antibodies were diluted in PBS to a final concentration of ~10 nM and adsorbed to the coated glass slides. Individual molecules were imaged under experimental conditions. Localizations originating from single Alexa 647 (A) and Alexa 750 (B) molecules were aligned to allow the estimation of the average localization precision: σx,y A647 = 12 nm and σx,y A750 = 21 nm. (PDF) [file ppat.1008656.s006.pdf]

A

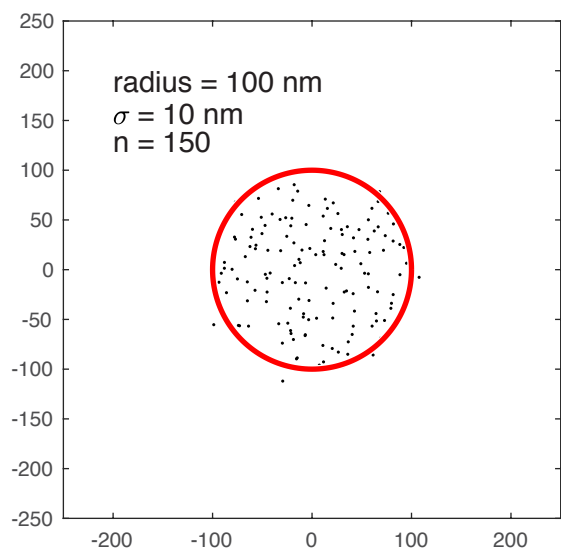

B

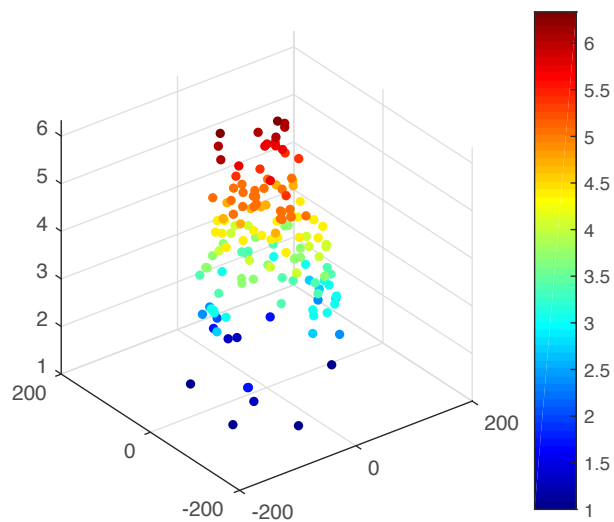

C

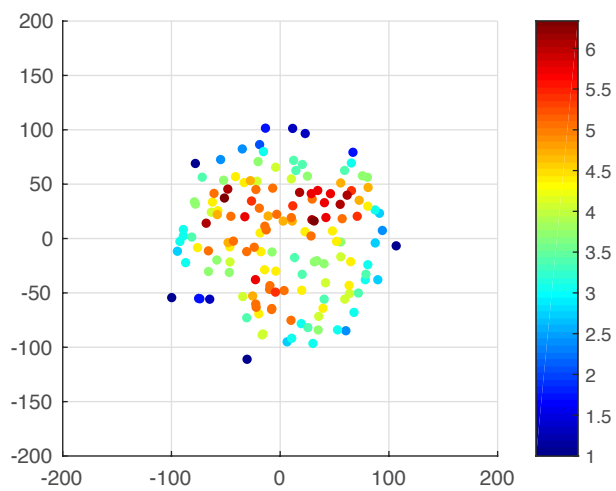

D

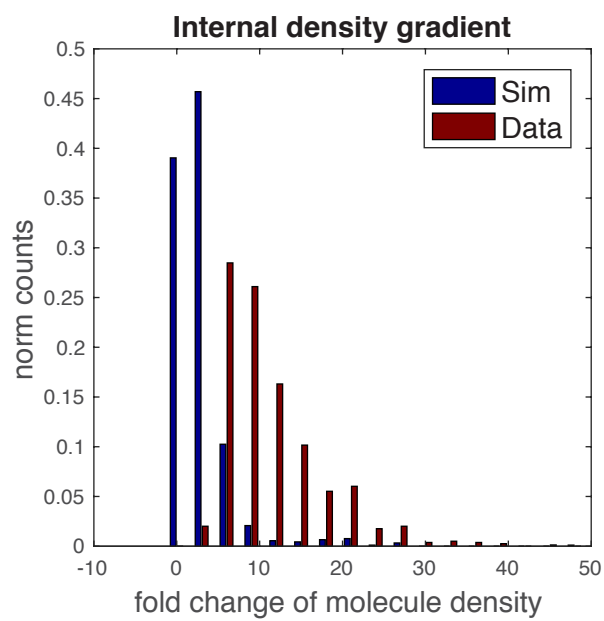

Supplement: S6 Fig — To test if the localization precision accounts for the gradient in localization density we observed in AF clusters (Fig 3), we simulated clusters of random localizations (A) using cluster size data taken from our experimental STORM measurements (i.e. radius r, number of localizations n, localization precision σ). The local density was then determined using a nearest neighbor search within a radius of 3σ. We indeed observed that the simulated clusters exhibit an up to about 8-fold local enrichment (see one example in A-C). We then simulated clusters following the full distribution of experimental data (i.e. radius r, number of localizations n). Comparing with the density gradient observed in our experimental data (D andFig 3), we find that both distributions are well separated and that the described effect only accounts for density changes < 8-fold. (PDF) [file ppat.1008656.s007.pdf]

**A**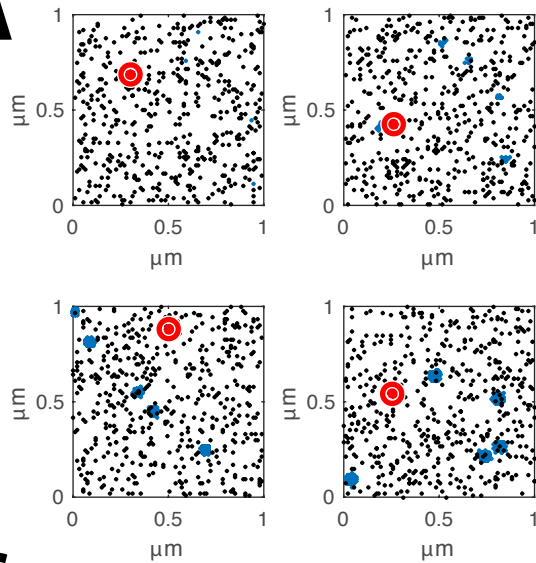**B**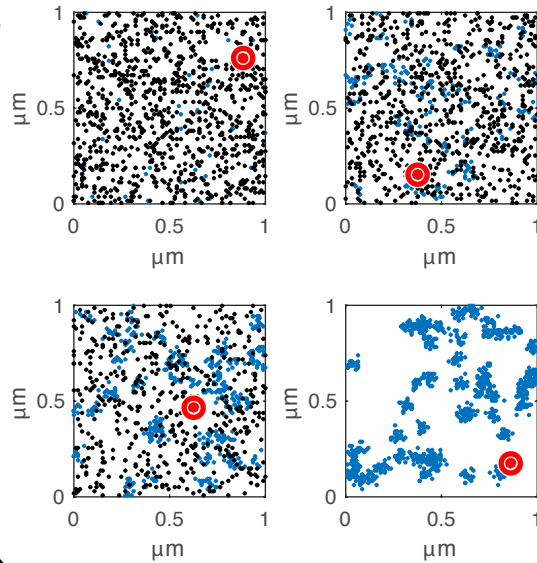**C**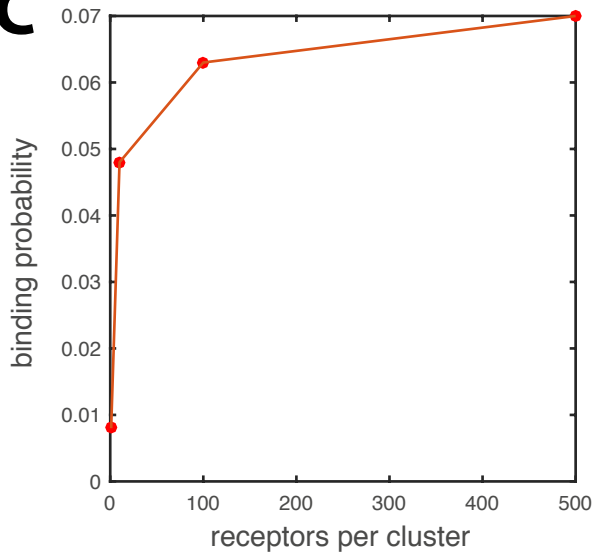**D**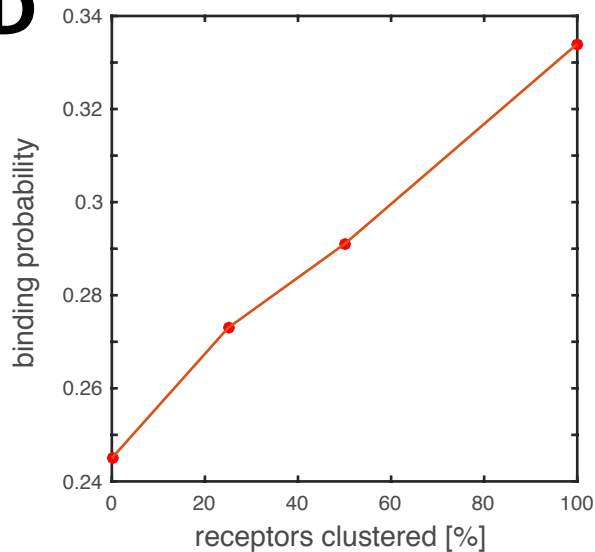

Supplement: S7 Fig — To estimate the effect of AF clustering on the efficiency of a virus to bind the target cell, we simulated two scenarios in a 1x1 μm membrane area, (A) a varying cluster size and (B) a varying degree of clustering. For A, we simulated a constant lateral concentration of AF (black) and added AF clusters (blue) at increasing size. In B, we keep the total amount of AF constant and gradually shift molecules into clusters. In both cases, an approaching virus was simulated as a 2D projection of a small spherical IAV particle (contact area as red circles in A and B). A binding attempt was counted as successful if at least 10 AF molecules were found inside the contact area. C and D show the simulation result plotted as the binding probability out of 1000 simulations against the respective tested cluster parameter. (PDF) [file ppat.1008656.s008.pdf]

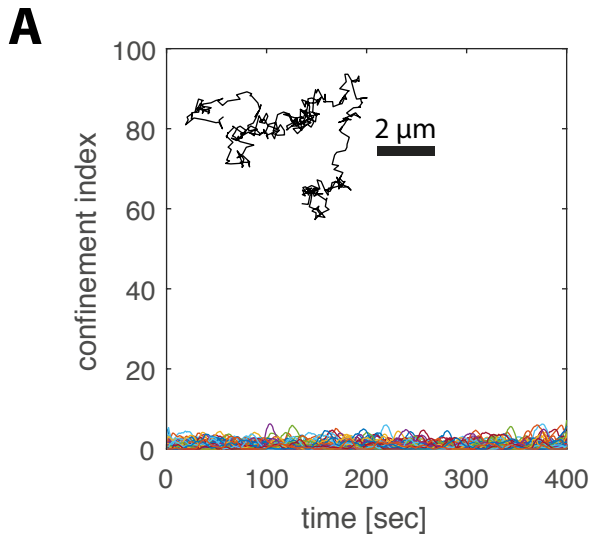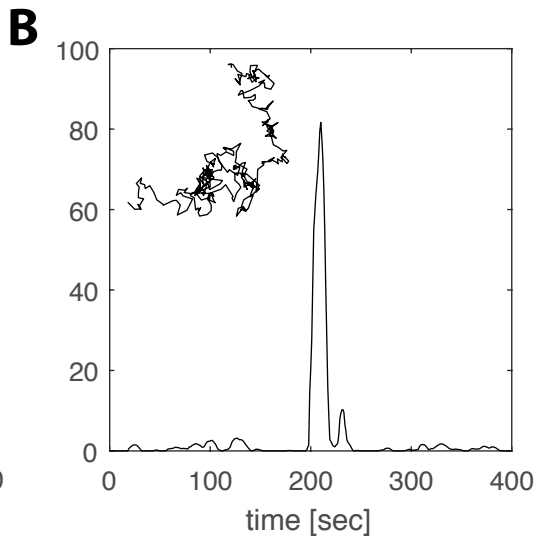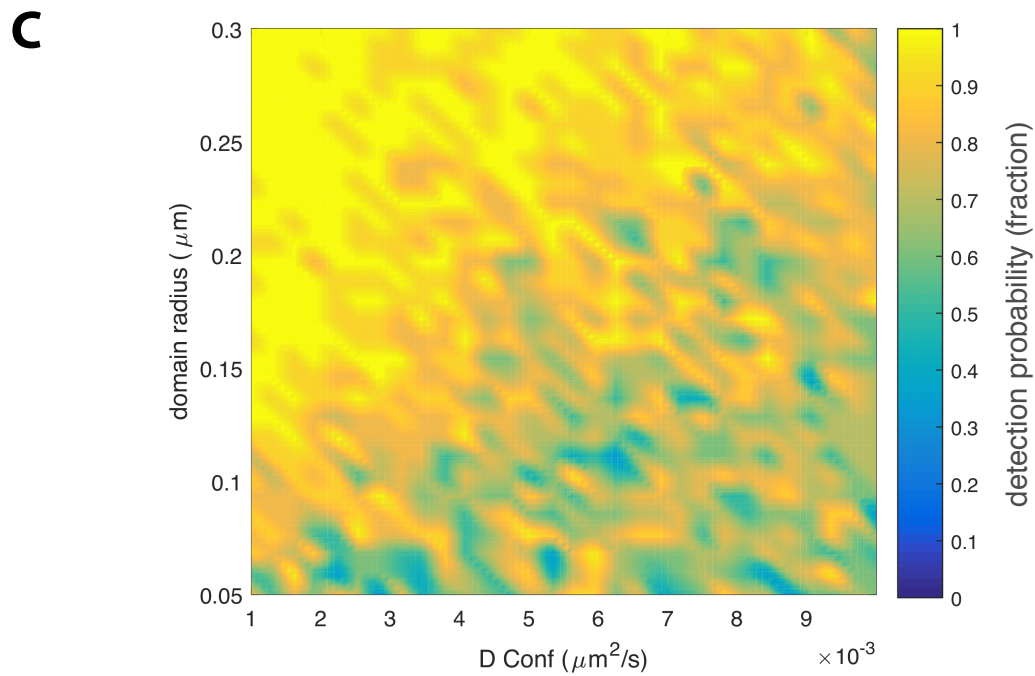

Supplement: S8 Fig — To test the precision of the confinement probability, we simulated random trajectories that did not or did contain a confined region and calculated the confinement probability Iconf for each trajectory. The confined region was chosen according to experimental data for the radius (r = 50–300 nm) as well as Dconf (Figs 2 and 3). To identify a confined region, we had to set a threshold of Iconf, which we set to 5 to allow high sensitivity. Our simulations (100 trajectories) show that at this threshold we have a chance of false identification (false positives) of ~10%, which drops to 0% at Iconf > 8 (A). For the simulations that did contain a confinement (example in B), we found an average detection efficiency (true positives) of 90% across the entire simulation space (900 trajectories, color code in C). (PDF) [file ppat.1008656.s009.pdf]

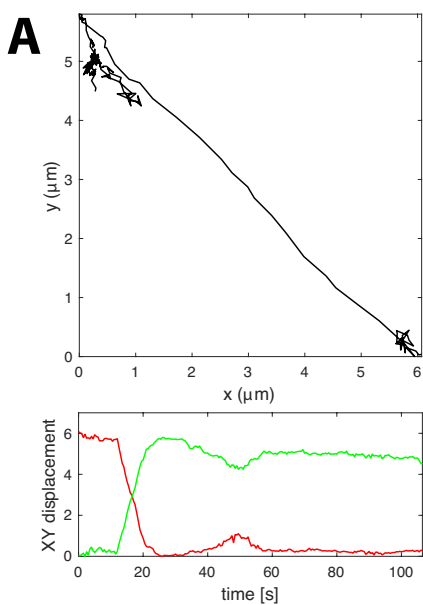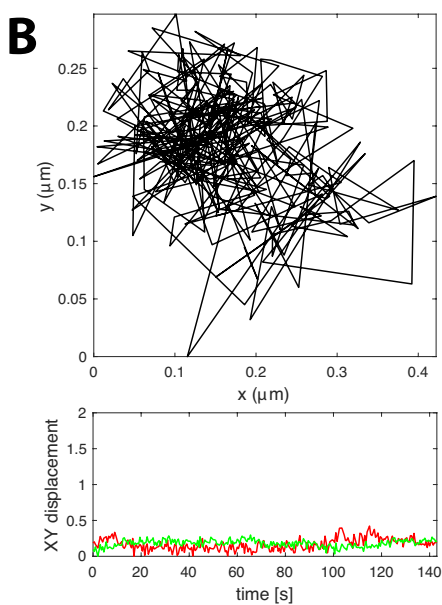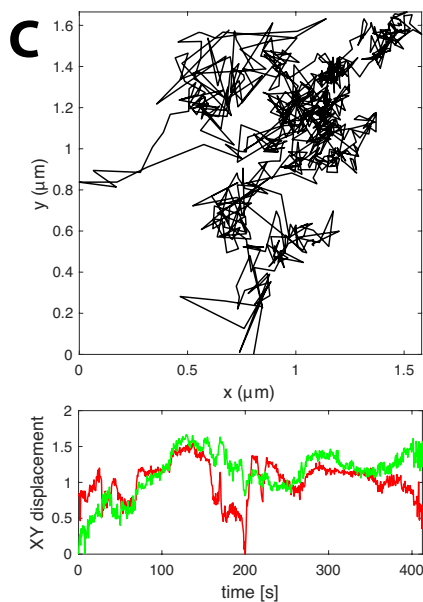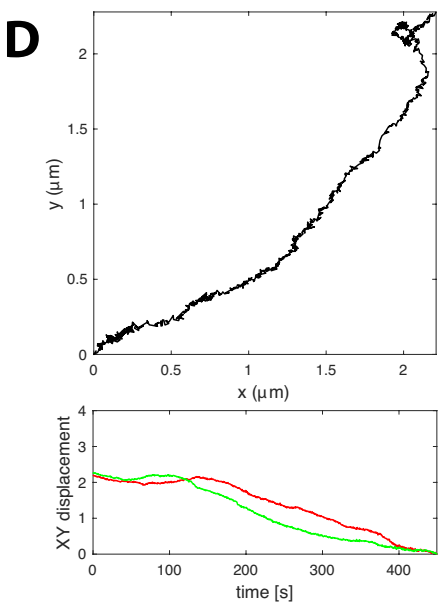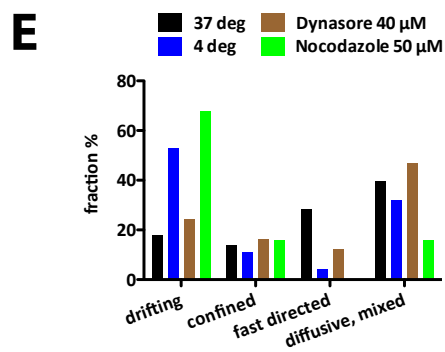

Supplement: S9 Fig — Single virus tracking on live A549 cells revealed four main types of virus movement: (A) three-stage movement, (B) confined, (C) mixed, (D) drift. The fraction of all modes of movement was analyzed at the indicated conditions (E). (PDF) [file ppat.1008656.s010.pdf]

**A**

Widefield

IAV (H3N2) / EGFR

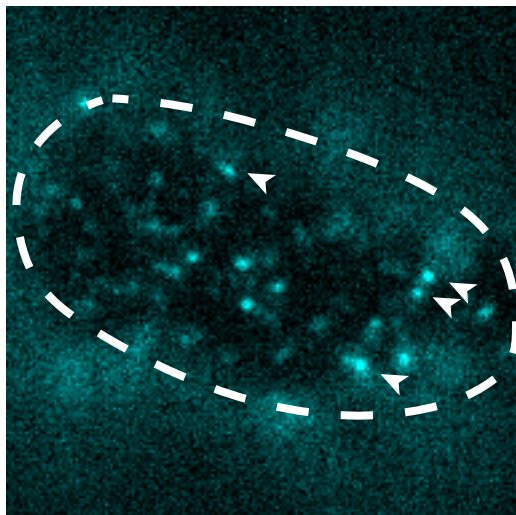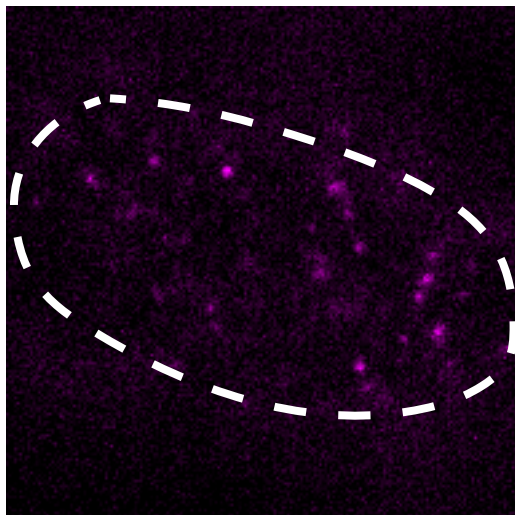

IAV (H3N2) / pEGFR

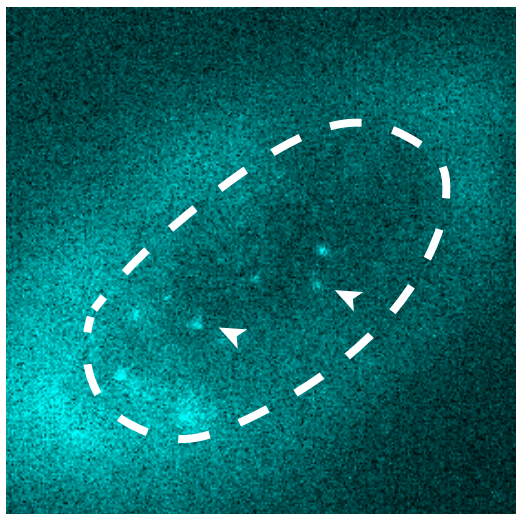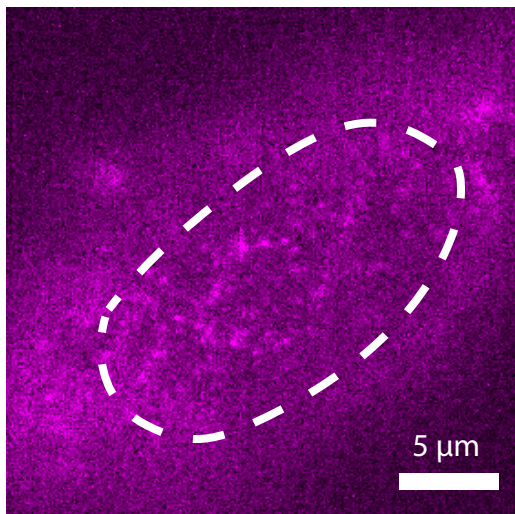**B**

STORM

IAV (H3N2) / EGFR

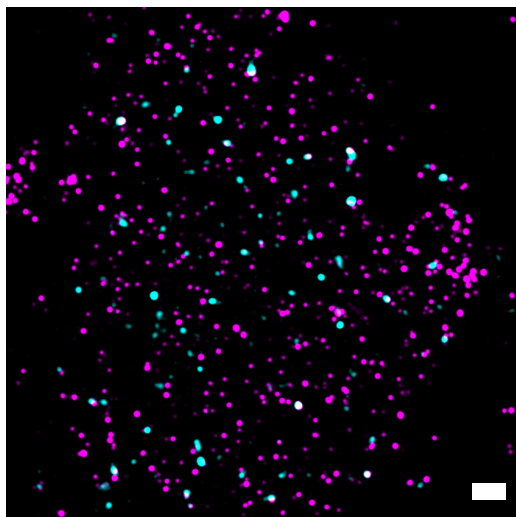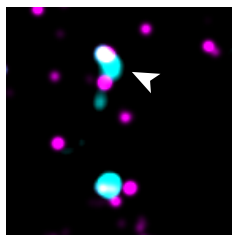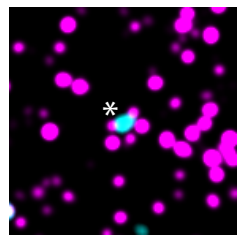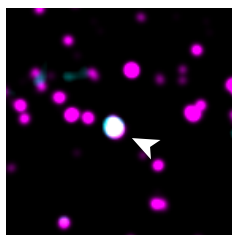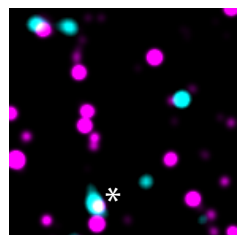

IAV (H3N2) / pEGFR

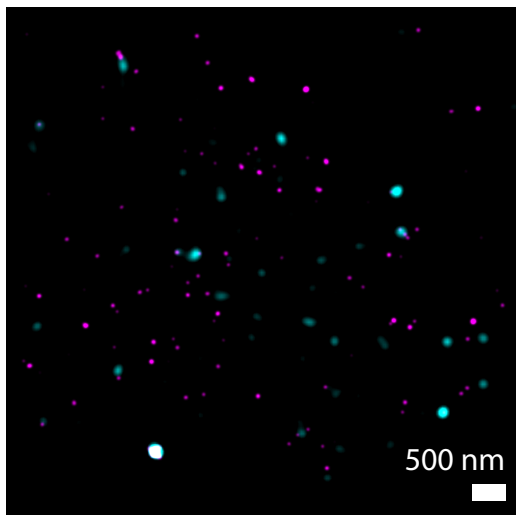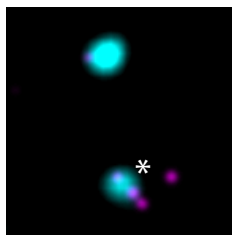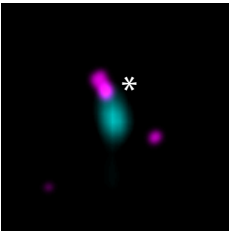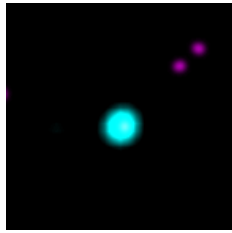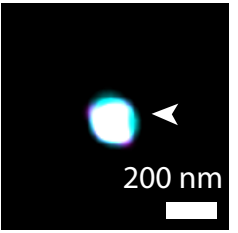

Supplement: S10 Fig — A549 cells were infected with IAV X31 (MOI = 100) at low temperature for 15 min, then fixed and immunostained using anti-H3N2 antiserum together with either anti-EGFR or anti-pEGFR (Y1068) antibodies. We analyzed images takes in widefield epi illumination (A) as well as two-color STORM (B). Using widefield epi illumination, we observed bright IAV spots that co-localize with EGFR clusters and pEGFR signal (arrow heads). Scale bar = 2 μm. We quantified this colocalization and found that in both cases about 20% of IAVs colocalize with EGFR and pEGFR respectively. In the two-color STORM reconstructions, we could also observe IAVs that colocalize with EGFR or pEGFR. More specifically, due to the increased resolution, we could discriminate fully overlapping colocalization (B, arrow heads) as well as IAVs that associate with more than one (p)EGFR cluster (B, asterisk). Shown are overview images (left) as well as four magnified regions (right) for each overview. (PDF) [file ppat.1008656.s011.pdf]

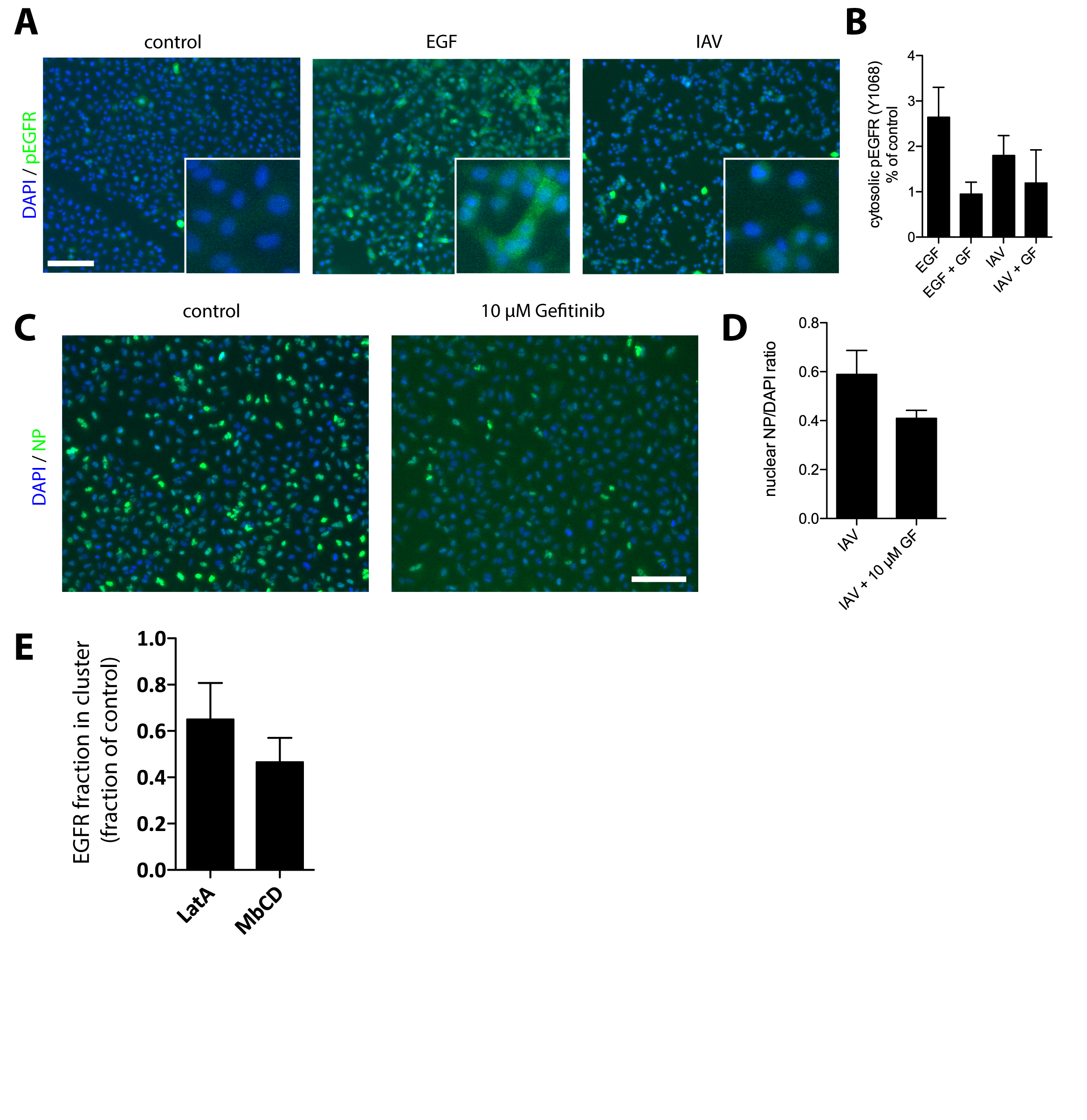

Supplement: S11 Fig — A549 cells were infected with IAV X31 (MOI = 100) at 4°C for 15 min upon pre-treatment with 10 μM Gefitinib for 1h min at 37°C. The cells were fixed and immunostained using pEGFR (Y1068) antibodies to label activated EGFR (A) and nuclear DNA was labelled with DAPI. The cytosolic pEGFR signal was analyzed using ImageJ (B). To probe the infection efficiency, A549 cells were infected with IAV X31 (MOI = 1) for 8h upon pre-treatment with 10 μM Gefitinib for 1h min at 37°C. The cells were fixed and immunostained using anti-NP antibodies (A) and nuclear DNA was labelled with DAPI (C). The infected was quantified as nuclear NP/DAPI signal as analyzed with CellProfiler (D). EGFR nanoclusters are sensitive to actin- or lipid domain destabilization (E). A549 cells were incubated with latrunculin A (1 μM) or methyl-b-cyclodextrin (MCD) (40 μg/ml) for 1 h at 37°C, then fixed and stained using anti-EGFR antibodies. (TIF) [file ppat.1008656.s012.tif]

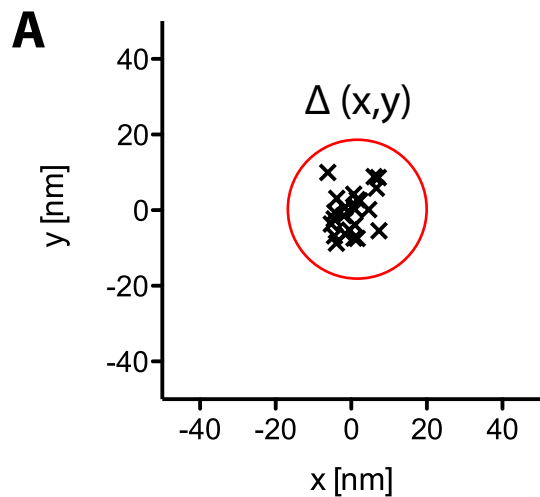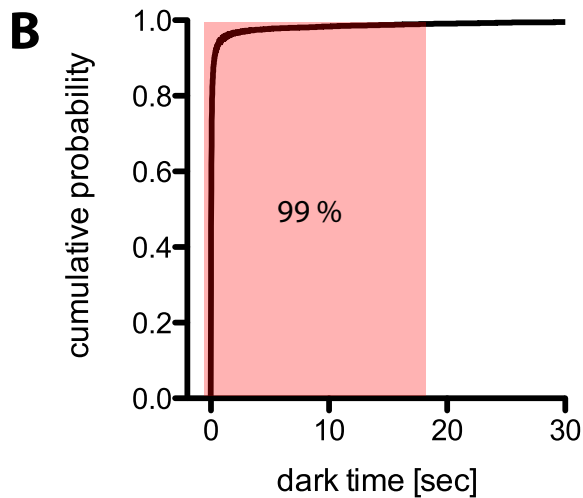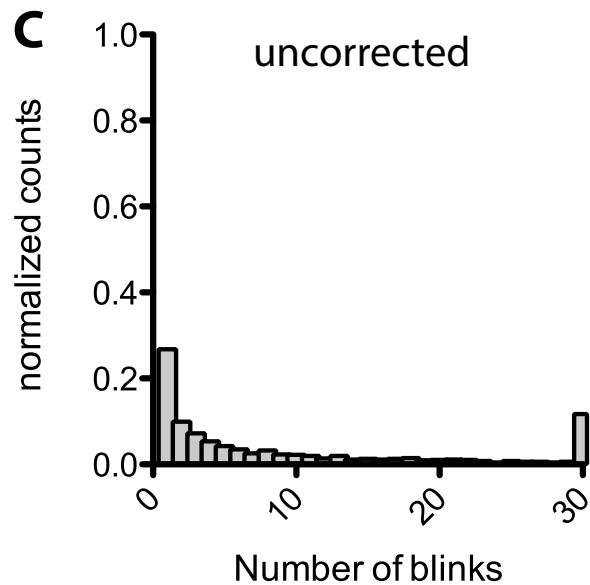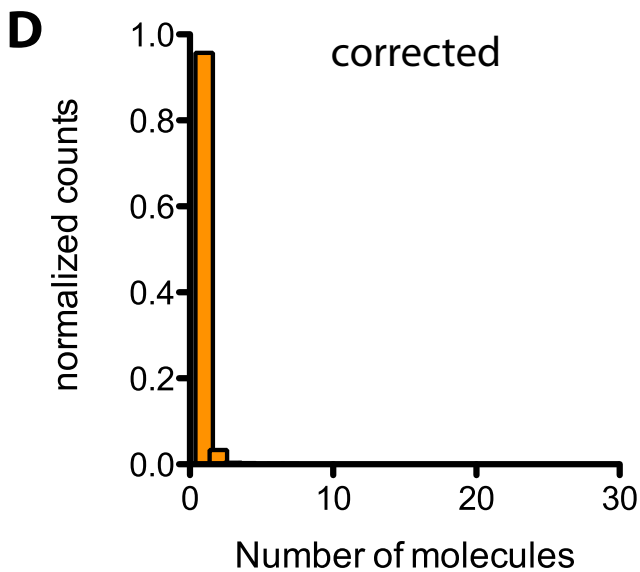

Supplement: S12 Fig — To estimate the number of emitting molecules from a STORM dataset and to avoid false clustering of individual molecules, we merged multiple localizations originating from the same molecule into a single localization. The merging procedure requires a gap distance as well as a gap time, within which localizations will be counted as originating from the same molecule. To calibrate these values, we imaged isolated labelled anti-EGFR antibodies under experimental conditions. Localizations originating from a single molecule could be grouped to determine their lateral spread (A) as well as the dark time between individual bursts (B). To ensure a high certainty of merging, the dark time cut-off was determined by the 99% quantile to 18 s. Using the experimentally determined spread of localization (A, 35 nm) and the dark-time cut off, localization bursts from the same molecule can now be combined into a single position. While each molecule is counted multiple times due to molecule blinking (uncorrected, C), merging allows a more precise estimate of the molecule numbers while avoiding false clustering (corrected, D). (PDF) [file ppat.1008656.s013.pdf]

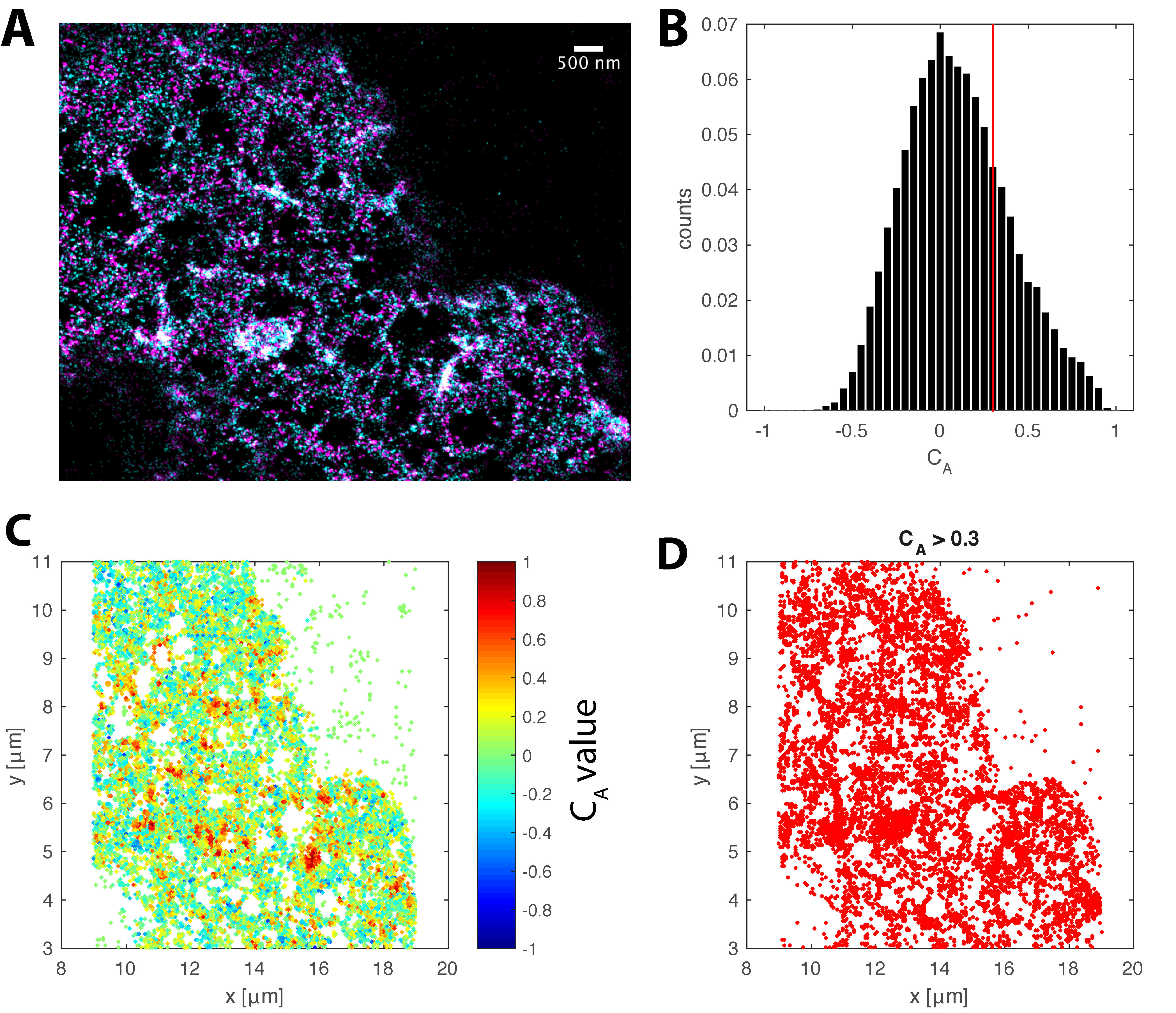

Supplement: S13 Fig — We used two differently labelled versions of SNA as an experimental colocalization positive control. This served us also as a nominator to better evaluate the degree of colocalization of our test molecule pair SNA/EGFR. A549 cells were labelled with two SNA variants, conjugated to Alexa 647 as well Alexa 555 (A). Both localization datasets were analyzed using CBC resulting in a colocalization value CA associated to each individual localization. A histogram of CA for one channel is shown in B. We set the threshold to 0.3, above which localizations were counted as colocalized. C shows one SNA dataset color coded according to CA. D shows all localizations from the same dataset with CA > 0.3. (PNG) [file ppat.1008656.s014.png]

**A**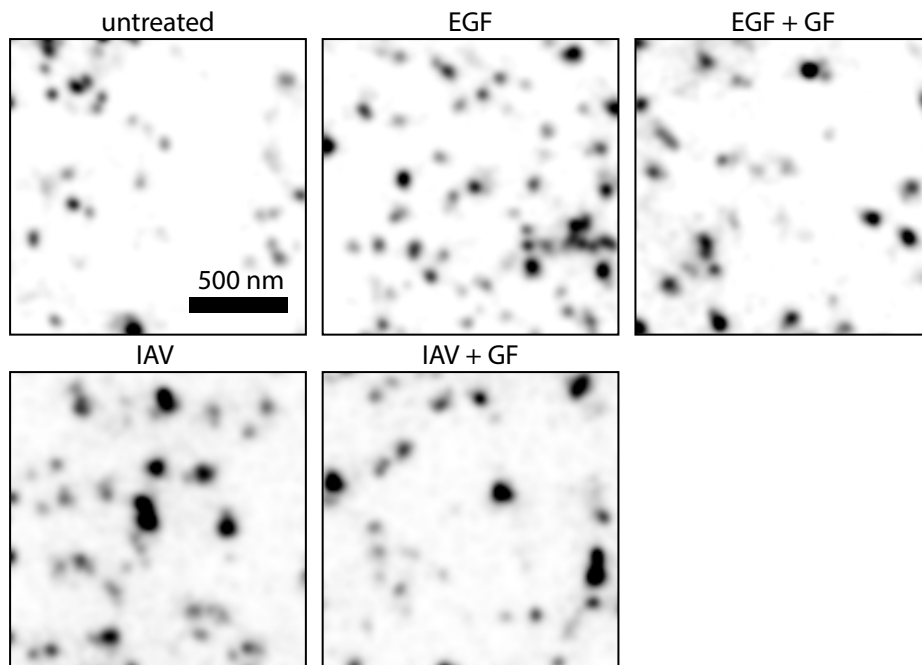**B**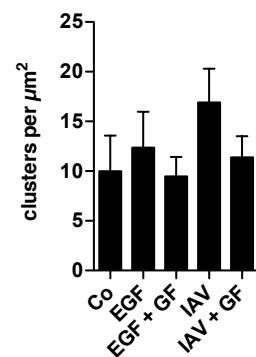**C**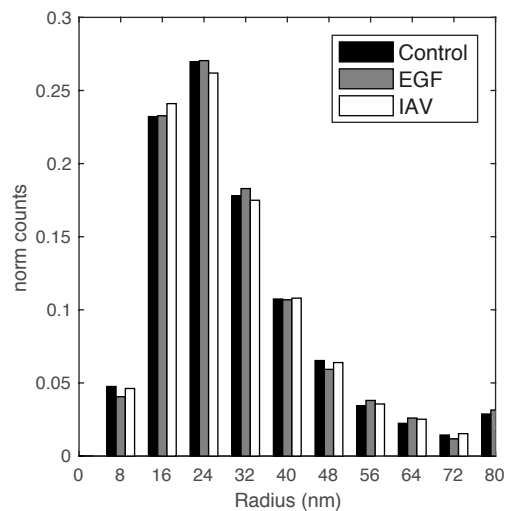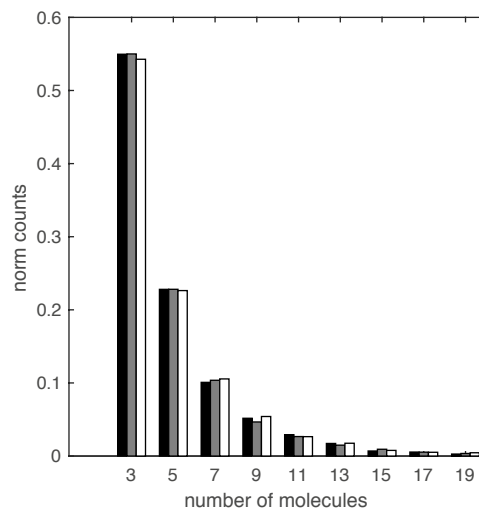

Supplement: S14 Fig — EGFR cluster activation can be inhibited by the EGFR kinase inhibitor Gefitinib (A, B). A549 cells were infected with IAV X31 (MOI = 100) at 4°C for 15 min upon pre-treatment with 10 μM Gefitinib for 1h min at 37°C. The cells were fixed and immunostained using pEGFR (Y1068) antibodies to label activated EGFR. The cells were imaged using STORM and pEGFR cluster identified using DBSCAN. Following stimulation with EGF or IAV, we detected an increase in the number of pEGFR cluster per area (as also shown in Fig 5). This effect could be inhibited by Gefitinib treatment (A, B). A549 were treated with infection medium (control) or infection medium containing IAV (MOI = 100) or 100 ng/ml EGF for 15 min at 4°C. The cells were fixed and immunostained using anti-EGFR antibodies. Upon either stimulation, we could not detect a change in the size of the EGFR clusters or the amount of molecules per cluster (C). (PDF) [file ppat.1008656.s015.pdf]

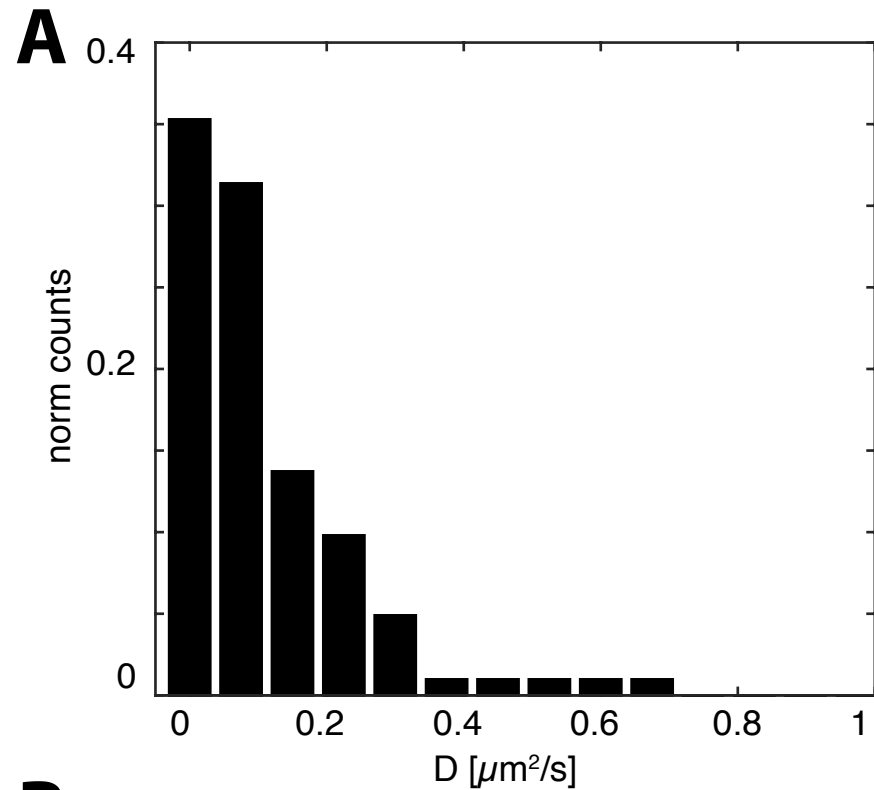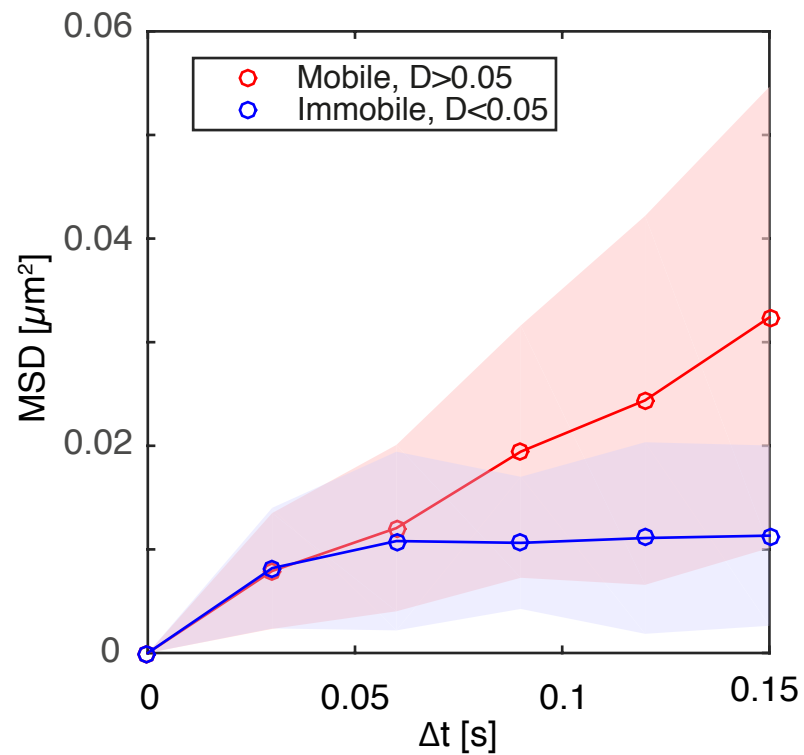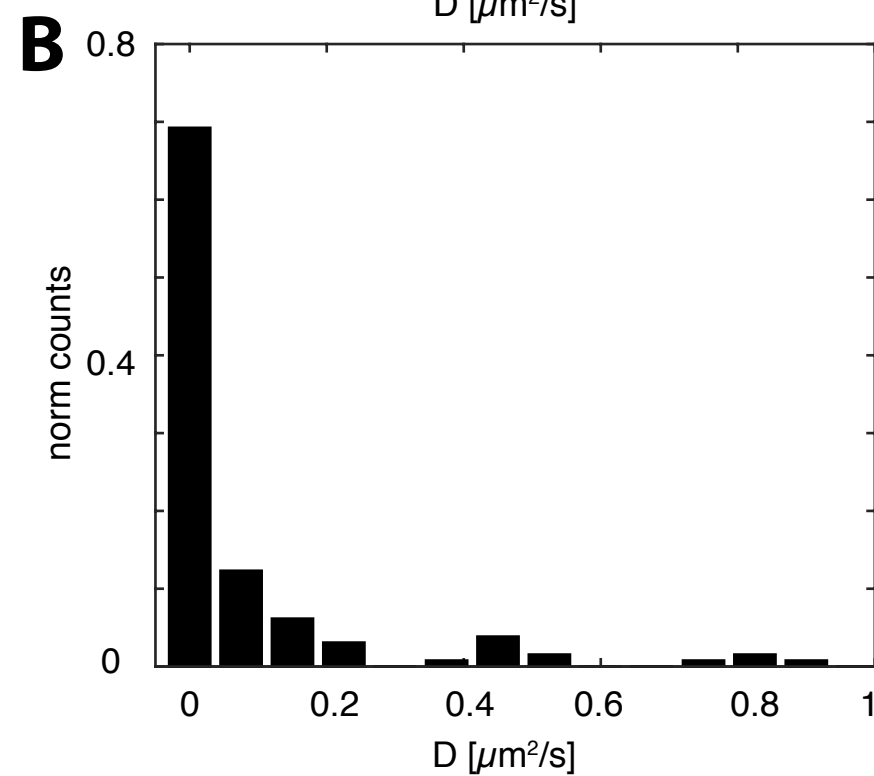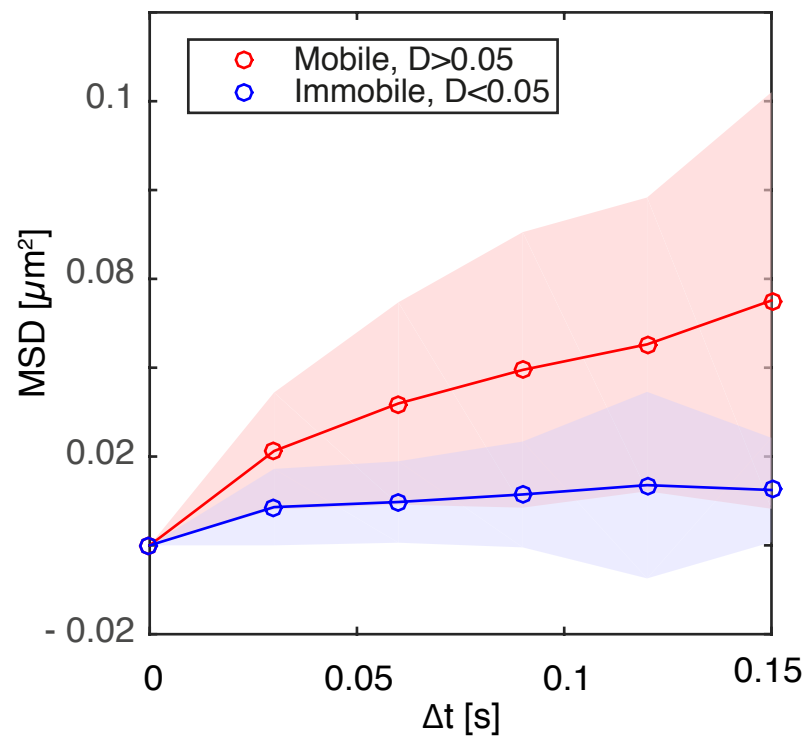

Supplement: S15 Fig — A549 cells were transiently transfected with EGFR-mEos3. sptPALM imaging and analysis of single molecule trajectories revealed a wide range of diffusion coefficients. The left panels in A and B show the distribution of diffusion coefficients from sptPALM obtained at the dorsal (A) and the ventral plasma membrane (B). Molecules were classified as mobile (D>0.5 μm2/s) or immobile (D<0.5 μm2/s) respectively. Calculated MDS plots (A and B, right panels) for both classes exhibit a rather linear dependence for the mobile fraction, while the curve saturates with increasing lag time for the immobile fraction, the latter indicating spatial confinement. (PDF) [file ppat.1008656.s016.pdf]

**A**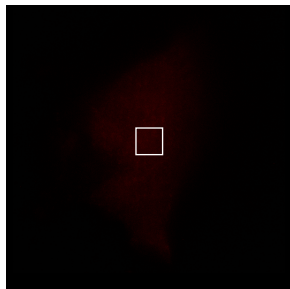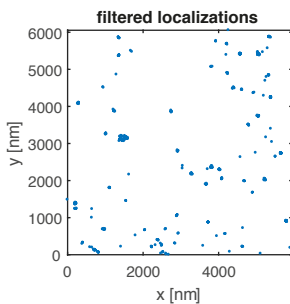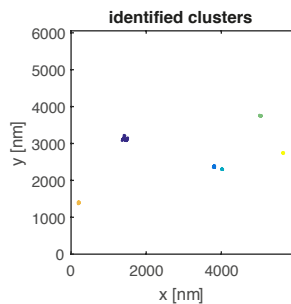

EGFR-mEos3.2

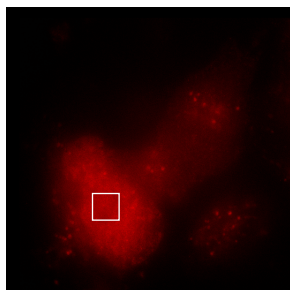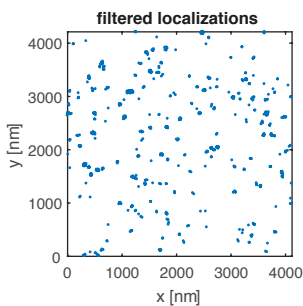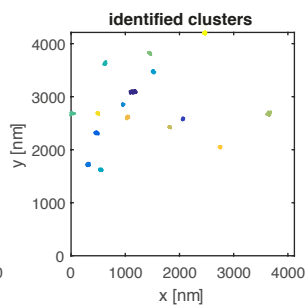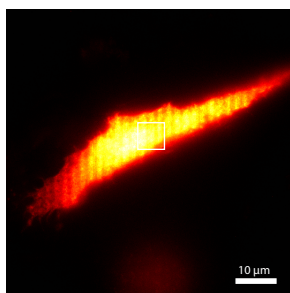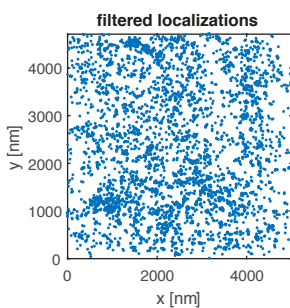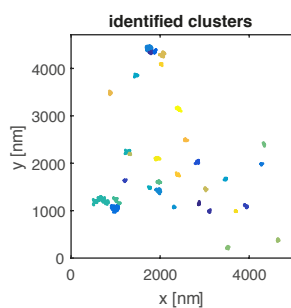**B**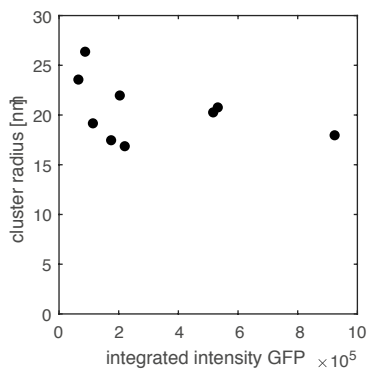

Supplement: S16 Fig — A549 cells were transfected with plasmids for EGFR-mEos3.2 24h before the imaging experiments. On the day of the experiment, the cells were fixed and and imaged. In order to assess the expression level, we took a snapshot of the pre-converted green mEos version before photoconversion and PALM acquisition. We performed the DBSCAN cluster analysis for different cells at various expression levels (A). We observed that the number of clusters per area increased at higher expression level, while the cluster radius was not affected (B). (PDF) [file ppat.1008656.s017.pdf]
